# An HMA-like integrated domain in the wheat tandem kinase WTK4 recognises an RNase-like pathogen effector

**DOI:** 10.1038/s41467-026-74940-x

**Published:** 2026-07-06

**Authors:** Zoe Bernasconi, Ursin Stirnemann, Yang Xu, Aline G. Herger, Renjie Chen, Victoria Widrig, Matthias Heuberger, Mariantonietta Lettieri, Marion C. Müller, Brande B. H. Wulff, Thomas Wicker, Beat Keller, Javier Sánchez-Martín

**Affiliations:** 1 Department of Plant and Microbial Biology, University of Zürich, Zürich, Switzerland; 2 Plant Science Program, Biological and Environmental Science and Engineering Division (BESE), King Abdullah University of Science and Technology (KAUST), Thuwal, Saudi Arabia; 3 Department of Microbiology and Genetics, Spanish-Portuguese Agricultural Research Centre (CIALE), University of Salamanca, Salamanca, Spain; 4 Research Unit of Excellence “Agricultural Production and Environment” (AGRIENVIRONMENT), University of Salamanca, Salamanca, Spain; 5 Present Address: Chair of Phytopathology, TUM School of Life Sciences, Technical University of Munich, Freising, Germany

**Keywords:** Plant molecular biology, Fungal genes, Agricultural genetics, Protein structure predictions

## Abstract

Proteins with a tandem kinase structure have recently emerged as players in race-specific resistance in cereal crops. However, the molecular understanding of these immune receptors’ resistance mechanisms is limited by the lack of knowledge about the pathogen effectors that they recognise. In this work, we identify AvrWTK4, the wheat powdery mildew RNase-like effector recognised by the wheat tandem kinase protein WTK4, through a combination of bi-parental genetic mapping and mutagenesis. We demonstrate that mutations in the *AvrWTK4* gene or a reduction of its expression lead to virulence on *WTK4*. Transfection of *AvrWTK4* specifically induced cell death in *WTK4*-expressing *Aegilops tauschii* protoplasts. The avirulent AvrWTK4 variant interacts more strongly than the virulent variant with the N-terminal heavy metal-associated (HMA)-like domain of WTK4. We found that the integrated HMA-like domain possibly serves as a direct effector-binding decoy for WTK4, providing a mechanistic paradigm for effector recognition by tandem kinase proteins.

## Abstract (web summary)

The wheat powdery mildew avirulence effector AvrWTK4 is recognised by the integrated HMA-like domain of the tandem kinase resistance protein WTK4. Wheat powdery mildew evades from WTK4-mediated recognition through AvrWTK4 mutation or downregulation.

## Introduction

Cereal crops such as wheat and barley are essential components of the human diet, yet their production is threatened by pathogens that reduce agricultural yields^1^. Plants have an innate immune system composed of different layers; the first layer is activated by the recognition of conserved pathogen-associated molecular patterns (PAMPs) via membrane-bound receptors, leading to PAMP-triggered immunity (PTI)^2^. Adapted pathogens have evolved mechanisms to bypass PTI by secreting effectors, which repress the immune response and facilitate infection. However, plants have a second layer of defence, activated via recognition of effectors, then called avirulence (Avr) factors, by intracellular resistance (R) proteins, resulting in effector-triggered immunity (ETI)^3,4^. ETI is race-specific and frequently results in hypersensitive response (HR) and cell death.

Most known *R* genes encode nucleotide-binding leucine-rich repeat receptors (NLRs)^5^. In addition, kinase fusion proteins have recently been identified as a class of R proteins involved in ETI in wheat and barley^6,7^. These non-canonical *R* genes encode proteins with a domain organisation made of a kinase domain fused to either another kinase domain, forming what is known as tandem kinase proteins (TKPs) (some examples being *Rpg1*, *Yr15* = WTK1*, Sr60* = WTK2*, Pm24/RWT4* = WTK3*, WTK4* and *Sr62* = WTK5), and/or to domains of diverse nature (for example, *Pm4*) and have important roles in conferring race-specific resistance to various biotrophic fungal diseases, such as different rust species, powdery mildews and blast^7,8^.

Some NLR immune receptors carry integrated domains (IDs), which act as 'effector traps' by mimicking host targets of pathogen effectors^9,10^. By resembling these native targets, IDs enable direct interaction with effectors that manipulate host metabolism or immunity^10–12^. Well-characterised examples include the rice NLRs RGA5 and Pikp-1, both of which feature heavy metal-associated (HMA)-like IDs that bind the rice blast effectors AVR-Pia/AVR1-CO39 and AVR-Pik, respectively^13–15^. Notably, HMA-like IDs have recently emerged as promising targets for engineering immune receptors with expanded effector recognition^16^. Interestingly, recent work has shown that over half of plant TKPs also carry IDs, suggesting they may function similarly to ID-carrying NLRs, by acting as decoys for pathogen effectors^17^.

In powdery mildew of wheat (*Blumeria graminis* f. sp. *tritici*; *Bgt*) and barley (*B. hordei*; *Bh*), numerous studies have focused on identifying and characterising *Avr* effectors recognised by NLR immune receptors. Notable examples of NLR-recognised Avr effectors include the AvrPm2 and AvrPm3 effectors in *Bgt*^18–20^, and the Avra effectors in *Bh*^21^, all exhibiting a typical RNase-like structure. Although effectors from *Bgt* and *Bh* display high sequence diversification, the RNase-like structure is highly conserved in these species^22^. So far, the only NLR-recognised Avr effector in *Bgt* that does not have an RNase-like structure is the recently described AvrPm3e_1^23^. Genetic approaches such as GWAS and bi-parental mapping have been instrumental in identifying *Avr* genes in both pathosystems^18,20,24,25^. Recently, UV mutagenesis led to the discovery of *AvrPm4*, a *Bgt* effector recognised by a kinase fusion protein^26^, as well as the avirulence regulator *Bgt-646*, whose disruption results in virulence on both the NLR Pm3a and the TKP WTK4, suggesting a shared regulatory pathway^27^. *Avr* effectors from both *Bgt* and *Bh* typically trigger cell death when co-expressed with their corresponding *R* gene in *Nicotiana benthamiana* or wheat/barley protoplasts^26,28,29^. In *Bh*, Avra effectors directly interact with their corresponding Mla receptors^30,31^. In *Bgt*, AvrPm3^b2/c2^ was recently found to associate with the LRR domain of Pm3b^32^, while a predicted wheat zinc finger protein has been identified as a mediator of the Pm2-AvrPm2 interaction, forming a tripartite complex essential for immune activation^33^.

Although kinase fusion proteins have been described as immune receptors mediating race-specific resistance, their underlying resistance mechanisms remain poorly understood^34,35^. Among the few characterised examples, AvrPWT4, the *Magnaporthe oryzae* effector recognised by the TKP RWT4^36,37^, was recently shown to interact with RWT4 via a newly identified ID, formed by a partial duplication of the RWT4’s kinase domain, KDup^38^, suggesting a possible role for IDs as effector decoys in TKPs. Furthermore, we recently demonstrated that AvrPm4 directly interacts with, and is phosphorylated by, the kinase fusion protein Pm4^26^. Recent studies indicate that TKP-mediated resistance may depend on additional host components. For instance, both SR62 and RWT4 require a helper NLR to activate immune responses in the presence of avirulent AvrSr62 and AvrPWT4 variants, respectively^39,40^. Despite these advances, our understanding of how pathogen effectors evade recognition by TKP immune receptors is limited. Identifying these Avr effectors and investigating how they evolve to evade TKP-mediated recognition are key steps towards evaluating resistance durability and guiding the development of more effective and sustainable resistance strategies^41,42^.

In this study, we combined bi-parental mapping and UV mutagenesis to identify *AvrWTK4*, encoding an RNase-like effector of wheat powdery mildew recognised by the TKP WTK4. We showed that point mutations and modulation of *AvrWTK4* expression, mediated by the deduced ankyrin-repeat-containing protein Bgt-646, are the main mechanisms to overcome *WTK4*-mediated resistance. We identified an HMA-like ID at the N-terminus of WTK4 and showed that it directly interacts with AvrWTK4. Modifications of the binding residues, either in the HMA-like ID or in AvrWTK4, led to reduced interaction. Our findings reveal a TKP which mediates resistance through an integrated decoy mechanism. This may represent an evolutionarily conserved strategy among tandem kinase immune receptors and potentially offers a framework for engineering these immune receptors in cereals to gain expanded recognition specificity.

## Results

### Bi-parental mapping in wheat powdery mildew identifies a single *AvrWTK4* locus on chromosome 5

To understand at the molecular level the resistance function of the previously cloned *R* gene *WTK4*^43^, we aimed at identifying its corresponding avirulence (*Avr*) gene by combining bi-parental genetic mapping and AvrXpose, a UV mutagenesis-based approach for *Avr* gene identification^27^.

**Fig. 1:**
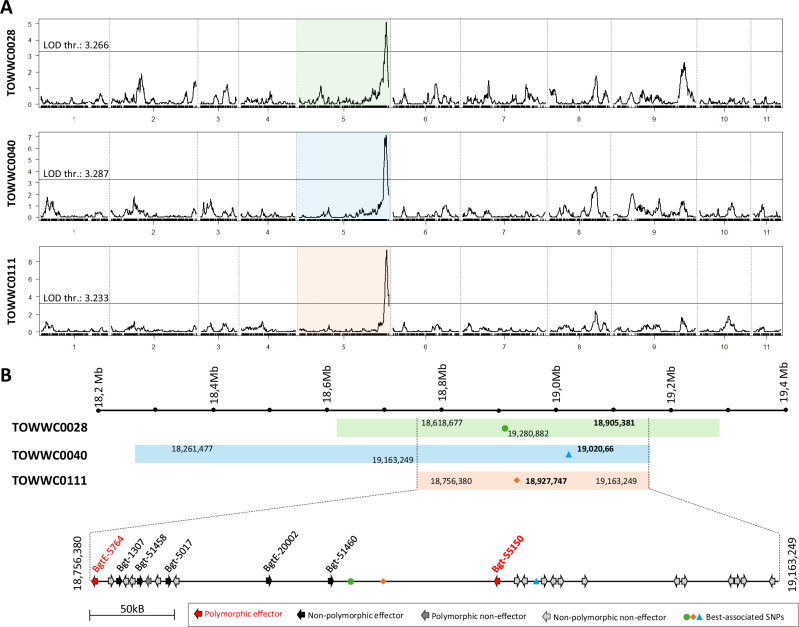
Identification of the *AvrWTK4* locus using a bi-parental mapping population. **A** QTL mapping in a bi-parental mapping population (CHE_96224 x THUN-12^44^) on three different *Ae. tauschii* lines containing *WTK4* identified a single locus for avirulence on *WTK4* on chromosome 5 (coloured). The LOD (logarithm of the odds) threshold, shown as a black line, was calculated using 1000 permutations. **B** The locus interval varied between the three different *Ae. tauschii* lines (TOWWC0028, TOWWC0040 and TOWWC0111); however, their regions of best association overlapped. The best-associated SNPs for the *Ae. tauschii* lines are located within the common region of association, between 18,756,380 and 19,163,249 Mb in chromosome 5 of the CHE_96224 genome (*AvrWTK4* locus). These SNPs are represented as a dot (TOWWC0028), a triangle (TOWWC0040), and a square (TOWWC0111). The LOD scores at the position of the best-associated SNP were 5.086 (TOWWC0028), 7.135 (TOWWC0040) and 9.291 (TOWWC0111). Among the 25 annotated genes within the *AvrWTK4* locus, seven are annotated as effectors^44^, and two of them, *BgtE-5764* and *Bgt-55150*, are polymorphic between CHE_96224 and THUN-12 (indicated with a red arrow; see also Supplementary Data 2).

For bi-parental mapping, we used a pre-existing F1 mapping population derived from a cross between a *Bgt* isolate, CHE_96224, and a *B. g. triticale* isolate, THUN-12^44^. We observed that THUN-12 was virulent on 21 *Ae*. *tauschii* lines containing *WTK4*, on which CHE_96224 was avirulent (Supplementary Data 1^43^). This indicated that THUN-12 did not harbour *AvrWTK4*, making the CHE_96224 x THUN-12 mapping population^44^ suitable to map the *AvrWTK4* locus. Three *Ae. tauschii* lines, TOWWC0028, TOWWC0040 and TOWWC0111, were randomly selected from the 21 lines mentioned above, and 83, 70 and 70 randomly chosen progeny of the *Bgt* mapping population were used for phenotyping on the three lines, respectively (Supplementary Data 2). For all three lines, a single locus for avirulence on *WTK4* was identified on chromosome 5 (Fig. 1A). The locus interval varied between the three different *Ae. tauschii* lines used; however, the region of best association was overlapping (18,756,380 Mb to 19,163,249 Mb; hereafter, *AvrWTK4* locus) and contained the best associated SNP for all three lines (Fig. 1B). In the *AvrWTK4* locus, there were 25 annotated genes, among which three were polymorphic between THUN-12 and CHE_96224. Two of these three genes, *Bgt-55150* and *BgtE-5764*, were annotated as effector genes, encoding proteins with a predicted signal peptide (Fig. 1B and Supplementary Data 3). On the protein level, Bgt-55150^THUN-12^ differs from Bgt-55150^96224^ by three amino acids (T30N, T33P and E35Q), while BgtE-5764^THUN-12^ differs from BgtE-5764^96224^ by one amino acid (V255A). Bgt-55150 is 119 amino acids in length and highly expressed, whereas BgtE-5764 is 257 amino acids in length and expressed at a low level at the haustorial stage in CHE_96224^45^. Based on these characteristics, we considered *Bgt-55150* the most likely *AvrWTK4* candidate gene. Interestingly, the previously cloned gene *AvrPm3*^*b2/c2*^ (*BgtE-20002*) is also in the *AvrWTK4* locus; however, it is not polymorphic between THUN-12 and CHE_96224, which are both avirulent on *Pm3b*^46,47^. Therefore, it was not considered for further investigation.

### *WTK4* gain-of-virulence mutants have mutations in the *Avr* candidate *Bgt-55150*

**Fig. 2:**
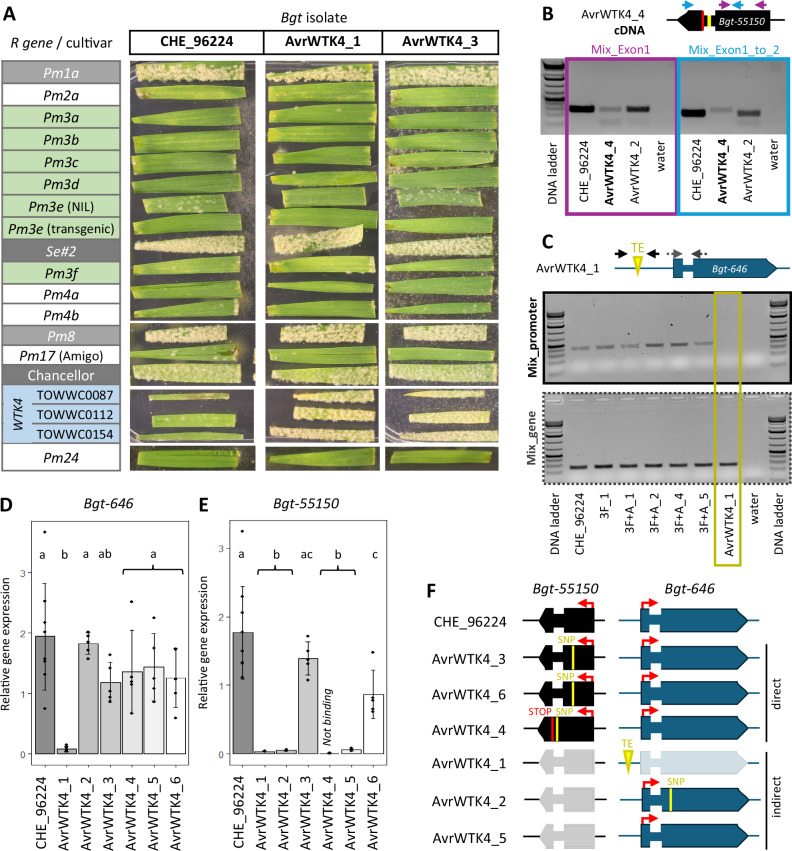
The *AvrWTK4* mutants exhibit direct or indirect gain-of-virulence mechanisms, all affecting *Bgt-55150.* **A** Wheat lines containing different *R* genes were infected with CHE_96224 and the *AvrWTK4* mutants. Cultivars susceptible to CHE_96224 (the *Pm1a* near-isogenic line (NIL), the sister line of the transgenic *Pm3e* line (here called *Se#2*), the *Pm8* NIL and Chancellor) are highlighted in grey. NILs containing *Pm3* alleles are depicted in green, and the three *Ae. tauschii* lines containing *WTK4* are in blue. The remaining *Pm* lines are shown in white. The phenotype of two representative mutants, AvrWTK4_1 and AvrWTK4_3, is shown. **B** PCR amplification at the cDNA level of *Bgt-55150* shows an increased size of the central gene part (Mix_Exon1_to_2) in AvrWTK4_4 compared with CHE_96224 and AvrWTK4_2 (220 base pairs instead of the 165 expected), indicating that the intron is retained. **C** PCR amplification of the *Bgt-646* gene (grey dashed arrows) and promoter region (black arrows) in CHE_96224, five selected 3F and 3F+A mutants (virulent on *Pm3a* and *WTK4*; see previous study^27^) and mutant AvrWTK4_1. No amplification was observed for AvrWTK4_1 at the site of the TE insertion, indicating that the TE insertion is too large for amplification under the extension time used. The drawing of *Bgt-646* is not to scale. Expression analysis of **D** *Bgt-646* and **E** *Bgt-55150* in the *AvrWTK4* mutants. One of the primers for *Bgt-55150* detection spans over the two exons (Supplementary Data 6). Thus, for mutant AvrWTK4_4, where splicing is not occurring due to an intron mutation, the primer is not binding. The observed lack of relative gene expression confirms the intron retention. Five samples were measured for AvrWTK4 mutants and ten for CHE_96224. The whiskers show the standard deviation. Significance and *P* values (significance threshold of *P* < 0.05) were calculated using a two-sided analysis of variance (ANOVA) followed by a Tukey’s HSD test (Supplementary Data 7). Tests with the same letter are not significantly different. **F** Schematic representation of the genes *Bgt-55150* and *Bgt-646* in the *AvrWTK4* mutants. Direct or indirect changes in the sequence or expression of *Bgt-55150* are indicated. Brighter boxes indicate genes with low or no expression, while expressed genes are darker boxes marked with a red arrow. SNPs and TE insertions are in yellow. Gene representations are not to scale.

To confirm the *Avr* candidate found through bi-parental mapping as *AvrWTK4*, we employed the UV mutagenesis-based approach AvrXpose^27^ to generate *Bgt* gain-of-virulence mutants on *WTK4*. We generated six *Bgt* mutants virulent on *WTK4* (hereafter, *AvrWTK4* mutants), which were single-spore isolated and used for phenotyping on *WTK4*-containing *Ae. tauschii* lines (TOWWC0087, TOWWC0112 and TOWWC0154), as well as on a set of *Pm* differential lines to determine their virulence specificity. The *AvrWTK4* mutants were found to be virulent on the three tested *Ae. tauschii* lines containing *WTK4*, but not on any of the other tested *Pm* differential lines (Fig. 2A), except for mutant AvrWTK4_5, which was also virulent on *Pm3f* (Supplementary Fig. 1). The cause of this gain of virulence is currently unclear, since mutant AvrWTK4_5 does not have mutations in *AvrPm3*^*a2/f2*^ or *SvrPm3*, nor genes described as effector regulators, which could be linked to a *Pm3f*-virulence phenotype (Supplementary Data 4 and 5). Therefore, it is probable that an unknown mutation is responsible for the observed phenotype.

Whole genome sequencing and comparison to the non-mutated parental isolate CHE_96224 revealed that three *AvrWTK4* mutants (namely, AvrWTK4_3, AvrWTK4_4 and AvrWTK4_6) had a single nucleotide polymorphism (SNP) in the *AvrWTK4* candidate mapped above, *Bgt-55150*. Surprisingly, AvrWTK4_3 and AvrWTK4_6 shared the same mutation (T30N; Supplementary Data 4 and 5). These mutations were the result of independent events, as the mutants were isolated in separate mutagenesis experiments and showed a different set of additional mutations across the whole genome (Supplementary Data 4 and 5). Of note, the T30N mutation present in mutants AvrWTK4_3 and AvrWTK4_6 also occurs in the virulent variant of Bgt-55150 (Bgt-55150^THUN-12^), suggesting that this amino acid position is particularly relevant for specific recognition by WTK4. Mutant AvrWTK4_4 had a SNP in the predicted intron sequence of *Bgt-55150* (43 bp from the 5′ splice site and 13 bp from the 3′ splice site). We used primers binding on the two exons to PCR amplify a part of *Bgt-55150* from the cDNA of isolates CHE_96224 and AvrWTK4_4 and observed that the size of the PCR product was larger in AvrWTK4_4 (Fig. 2B), suggesting that the intron of *Bgt-55150* is retained. This was then confirmed by sequencing the PCR product. The loss of splicing leads to a premature stop codon in the translated protein. None of the *AvrWTK4* mutants had mutations in the coding sequence of *BgtE-5764*, the other polymorphic effector in the *AvrWTK4* locus found with bi-parental mapping (Supplementary Data 4 and 5). Mutant AvrWTK4_2 had a SNP in the putative 3′ regulatory region of *BgtE-5764*, 531 base pairs (bp) downstream of the stop codon; however, based on transcriptomic data of CHE_96224^45^ showing that the transcript level is very low at position 480 bp and absent at 670 bp after the stop codon of BgtE-5764, we concluded that the SNP has no effect on gene expression and that the causal mutation is likely affecting a different gene.

### Mutations in the effector regulatory gene *Bgt-646* result in a reduction of *AvrWTK4* expression and virulence on *WTK4*

As mutants AvrWTK4_1, AvrWTK4_2 and AvrWTK4_5 did not have mutations in *Bgt-55150*, we reasoned that additional components involved in avirulence on *WTK4* must exist. We investigated whether these three mutants had any commonly mutated genes and discovered that two of them had mutations affecting the gene *Bgt-646*, encoding a deduced ankyrin-repeat-containing protein. Mutant AvrWTK4_1 had an insertion of a transposable element (TE) 580 bp upstream of the start of the coding sequence (CDS) of *Bgt-646*, causing a strong reduction in *Bgt-646* expression (Fig. 2C, D), and mutant AvrWTK4_2 had a SNP in the CDS of *Bgt-646*, causing an amino acid change (R93K; Supplementary Data 4 and 5).

Interestingly, in a previous study, we observed an additional gain of virulence on *WTK4* in *Bgt* mutants with a gain of virulence on *Pm3a*, an NLR immune receptor^27^. Three of these previously described *Pm3a/WTK4* virulent mutants also had mutations in *Bgt-646*, which was then shown to regulate the expression of several effector genes in *Bgt*, including *Bgt-55150*^27^. To confirm whether this regulatory role extends to the *AvrWTK4* mutants, we performed an expression study and observed a significantly reduced expression of *Bgt-55150* in both *AvrWTK4* mutants with mutations in *Bgt-646*, suggesting that *Bgt-646* may affect *Bgt-55150* expression (Fig. 2E). Finally, mutant AvrWTK4_5 did not have mutations in *Bgt-646*, or any other commonly mutated gene, but *Bgt-55150* expression was also repressed (Fig. 2E). Thus, we hypothesise that additional, yet unidentified, mutations in AvrWTK4_5 reduce the expression of *Bgt-55150*, causing the gain of virulence. These findings further support our previous study^27^, indicating that mutations in *Bgt-646* result in lower expression of *Bgt-55150* and cause a loss of avirulence on *WTK4*.

In conclusion, all *WTK4*-virulent *Bgt* mutants have either direct (via SNPs) or indirect (via reduced expression) modifications of *Bgt-55150* (Fig. 2F). These data, combined with the bi-parental mapping approach, strongly indicate that *Bgt-55150* is *AvrWTK4*.

### *WTK4*-mediated resistance in *Ae. tauschii* induces a hypersensitive response

**Fig. 3:**
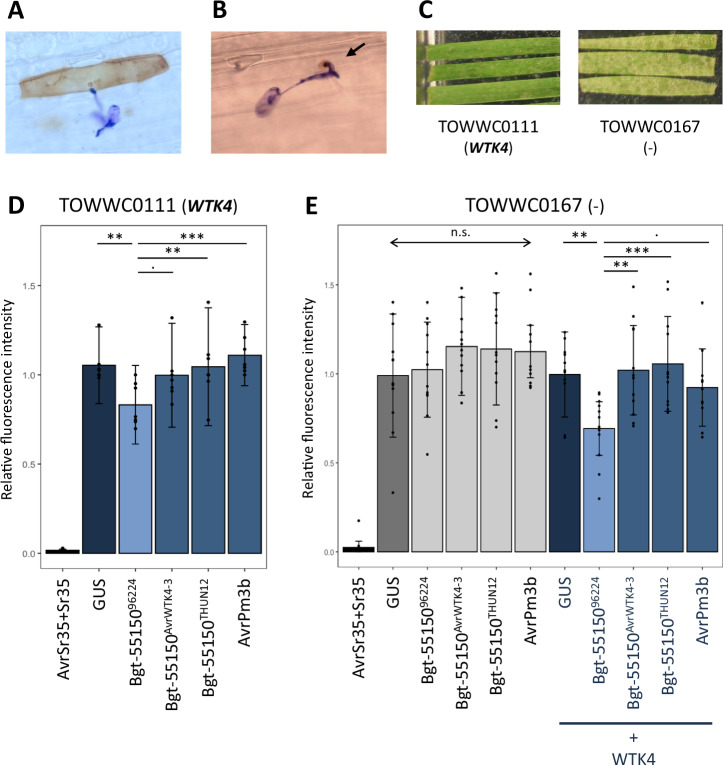
*WTK4*-mediated cell death in *Ae. tauschii* depends on *Bgt-55150*^*96224*^. **A** Example of a cell undergoing a hypersensitive response (brown colouring, visualised using DAB staining). **B** Example of a cell with papilla formation (round structure indicated by a black arrow). Both **A**, **B** represent microscopic evaluations of individual infection sites in a *WTK4*-containing line infected with CHE_96224 1 day after inoculation. The quantification of the different types of defence response is described in Supplementary Fig. 2. **C** The *Ae. tauschii* line TOWWC0111 (containing *WTK4*) is resistant, while TOWWC0167 is susceptible 7 days post inoculation with the *Bgt* isolate CHE_96224. **D** Protoplasts derived from TOWWC0111 and **E** TOWWC0167 were co-transfected with 3 pmol of a vector encoding YFP and 3 pmol of either *GUS*, *Bgt-55150*^*96224*^ (avirulent allele), *Bgt-55150*^*AvrWTK4_3*^, *Bgt-55150*^*THUN-12*^ (virulent alleles) or *AvrPm3*^*b2/c2*^ (negative control). For TOWWC0167, either *GUS* (grey bars) or *WTK4* (blue bars) was additionally transfected. The *Avr-R* pair *AvrSr35* + *Sr35* was used as a positive control. YFP fluorescence was measured 16–20 hpi for two technical and three biological replicates per treatment. The experiment was repeated three times for TOWWC0111 and four times for TOWWC0167. Fluorescence values were normalised to the average fluorescence of protoplasts co-transfected with the *YFP* vector and either *GUS* (TOWWC0111) or *WTK4* (TOWWC0167) for each experiment. The whiskers show the standard deviation. Significance and *P* values (significance threshold of *P* < 0.05) were calculated with a one-sided analysis of variance (ANOVA) followed by multiple comparisons of means (contrast analyses) with Bonferroni correction (***P ≤ 0.001; **P ≤ 0.01; *P ≤ 0.05; and •*P* ≤ 0.10; n.s. not significant; Supplementary Data 7).

NLRs that confer resistance to biotrophic pathogens, such as *Bgt,* in most cases, induce HR, leading to cell death and ultimately stopping the pathogen from spreading. To investigate whether the immune response mediated by *WTK4* also results in HR, we performed a microscopic evaluation of resistance upon infection. The avirulent isolate CHE_96224 was used to infect two *WTK4-*containing *Ae. tauschii* lines (TOWWC0028 and TOWWC0077), and the fungal infection was observed at 24, 48 and 96 hpi. In both *Ae. tauschii* lines, at least 25% of the spores attempting to penetrate were blocked by HR at all three timepoints (Fig. 3A and Supplementary Fig. 2). Papilla formation was also observed (Fig. 3B and Supplementary Fig. 2). These data provide evidence that the *WTK4*-induced immune response results in HR, very similar to other *Pm* resistance genes such as *Pm4*, encoding a kinase fusion protein^48^.

To functionally validate *Bgt-55150*^*96224*^ as *AvrWTK4*, we used a cell death assay relying on co-transfection of *Ae. tauschii* protoplasts, a method commonly used to functionally validate *Avrs* that trigger HR upon recognition^26,30,39,49^. The *Bgt-55150* gene sequences of CHE_96224 (avirulent), mutant AvrWTK4_3 and THUN-12 (both virulent) were codon-optimised for plant expression and cloned into expression vectors without a signal peptide. We selected *AvrPm3*^*b2/c2*^ as a negative control. It encodes an RNase-like effector which is most closely related to *Bgt-55150* at the sequence level. We selected a resistant (TOWWC0111, containing *WTK4*) and a susceptible (TOWWC0167, lacking *WTK4*) *Ae. tauschii* line for this experiment (Fig. 3C). Co-transfection of *Bgt-55150*^*96224*^ with a YFP marker in the *Ae. tauschii* line TOWWC0111 significantly reduced YFP fluorescence intensity compared to GUS, *Bgt-55150*^*THUN-12*^ and *AvrPm3*^*b2/c2*^ co-transfected with the YFP marker (Fig. 3D). In TOWWC0167, no significant difference in relative YFP fluorescence intensity was observed between protoplasts transfected with either GUS, *Bgt-55150*^*96224*^ or any other effector gene tested (Fig. 3E). However, when *WTK4* was additionally transfected in TOWWC0167, a significantly reduced YFP fluorescence intensity, indicative of cell death, was observed in combination with *Bgt-55150*^*96224*^ but not with the other tested effectors (Fig. 3E). These results indicate that *Bgt-55150*^*96224*^ induces cell death specifically in the presence of *WTK4* in *Ae. tauschii*. We therefore conclude that *Bgt-55150*^*96224*^ is *AvrWTK4*.

To check if all necessary components for the cell death response are conserved among other plant species, we performed cell death assays in *N. benthamiana* and in wheat protoplasts. Intriguingly, co-expression of *WTK4* with *Bgt-55150*^*96224*^ in *N. benthamiana* did not lead to cell death (Supplementary Fig. 3A). Because other TKPs, such as SR62^TK^ and RWT4, share the same helper NLR required for inducing HR in heterologous systems, we wondered whether *WTK4-Bgt-55150*^*96224*^ cell death could also be triggered by co-expression of this helper NLR (SR62^NLR^). We performed co-expression assays using the Fielder-derived *SR62*^*NLR*^ version, which successfully induced HR when co-expressed with *AvrSR62* and *SR62*^*TK*^. In contrast, co-expression of *WTK4-Bgt-55150*^*96224*^*-SR62*^*NLR*^ did not induce HR in *N. benthamiana*, indicating that SR62^NLR^ is not a helper of WTK4 (Supplementary Fig. 3A, B). In *N. benthamiana*, WTK4 accumulated at lower levels than the AvrWTK4 variants. It is possible that low protein levels could potentially limit its capacity to trigger strong HR. To complement the *N. benthamiana* experiments, we also performed a protoplast assay in wheat. Protoplasts of the wheat cultivar Fielder (which lacks *WTK4*) co-transfected with *WTK4* and *Bgt-55150*^*96224*^ did not show significantly reduced relative YFP fluorescence intensity compared to protoplasts co-transfected with *WTK4* and *GUS*, *Bgt-55150*^*AvrWTK4_3*^, *Bgt-55150*^*THUN-12*^ or *AvrPm3*^*b2/c2*^, indicating that no cell death occurred (Supplementary Fig. 3B). These findings indicate that at least one genetic component unique to *Ae. tauschii* and absent in *N. benthamiana* or the wheat cultivar Fielder is needed for inducing HR upon recognition of *Bgt-55150*^*96224*^, consistent with recent models where TKPs act as sensors that rely on helper proteins for full immune activation^39,40^.

### WTK4 interacts with AvrWTK4 through its HMA-like integrated domain

**Fig. 4:**
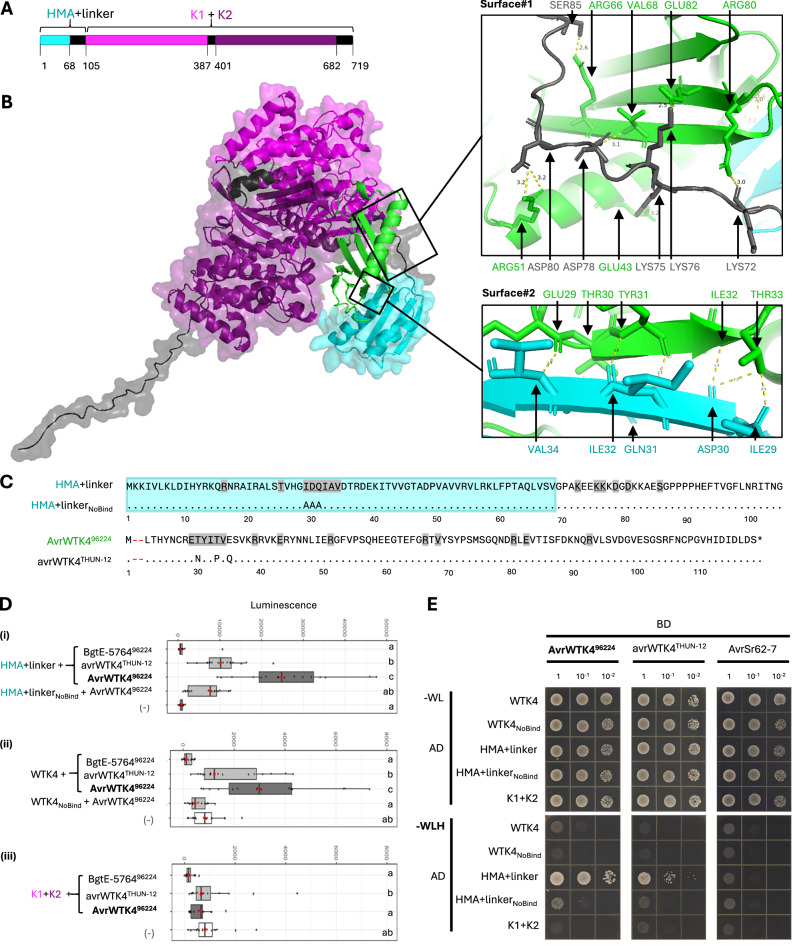
WTK4 interacts with AvrWTK4 through its HMA-like integrated domain. **A** Schematic view of WTK4 protein domains: HMA-like (cyan), pseudokinase (K1, light purple) and kinase (K2, deep purple) domains, and unstructured sequence (black). **B** AvrWTK4^96224^ (green), featuring an RNase-like fold (based on AlphaFold3 modelling), is predicted to interact with WTK4 through two main interaction surfaces: Surface#1, involving the linker sequence, and Surface#2, involving the HMA-like ID. Residues involved in each interaction, along with distances (in ångström), are shown in the corresponding images. **C** Protein sequences of the HMA-like ID with the linker sequence (HMA+linker), a modified version with three amino acid changes and reduced binding affinity to AvrWTK4^96224^ (HMA+linker_NoBind_), and two variants of Bgt-55150 (AvrWTK4^96224^ and avrWTK4^THUN-12^) are shown. Signal peptides are omitted (two red dashes). Residues predicted to be involved in interactions are highlighted in grey and underscored. **D** The interaction of AvrWTK4^96224^, avrWTK4^THUN-12^ and BgtE-5764^96224^ was tested with (i) HMA_WTK4_ fused to the linker sequence (HMA+linker), (ii) WTK4 full-length and (iii) the two kinase domains of WTK4 (K1+K2). avrWTK4^THUN-12^ and BgtE-5764^96224^ interacted less than AvrWTK4^96224^ with both the full-length WTK4 and the HMA+linker domain. The modified constructs WTK4_NoBind_ and HMA+linker_NoBind_ showed significantly reduced luminescence relative to their non-mutated versions. The negative control for all experiments is AvrWTK4^96224^ with the NLR protein PM17^25^. Sixteen biological and two technical replicates were analysed per treatment. In each box plot, the median is represented by the central line, and the box denotes the interquartile range (25th–75th percentile). The whiskers span between the most extreme data points within ±1.5 ×  interquartile range (IQR). Significance and *P* values (significance threshold of *P* < 0.05) were calculated with a two-sided analysis of variance (ANOVA) followed by a Tukey’s HSD test (Supplementary Data 7). Boxes with the same letter are not significantly different. **E** A yeast-2-hybrid (Y2H) assay shows significantly stronger interaction of AvrWTK4^96224^ with HMA+linker compared to avrWTK4^THUN-12^. AvrWTK4 variants were fused to the GAL4 binding domain (BD) and WTK4 variants to the GAL4 activation domain (AD). Yeast suspensions at an OD_600_ = 1.0 and two serial dilutions of 1:10 and 1:100 were spotted on synthetic medium lacking tryptophan and leucine (-WL, growth control) and on synthetic medium lacking tryptophan, leucine and histidine (-WLH, interaction selection). Immunoblot of each protein is shown in Supplementary Fig. 7. The negative control, AvrSr62-7, did not interact with any of the constructs tested.

To assess whether WTK4 and Bgt-55150^96224^ (hereafter, AvrWTK4^96224^) interact at the protein level, we first modelled their 3D protein structures using AlphaFold2 and AlphaFold3^50^. AvrWTK4^96224^ is predicted to exhibit the typical RNase-like fold of most *Bgt Avrs* identified to date (Fig. 4B)^29,46^, while WTK4, in addition to the pseudokinase (K1) and kinase (K2) domains, also has a predicted N-terminal HMA-like domain, which was not originally described^43^ (Fig. 4A, B; amino acid residues 1–68; hereafter HMA_WTK4_). HMA_WTK4_ shows relatively low amino acid identity to HMA-like IDs of known NLR proteins (i.e. RGA5 and Pikp-1; 30.8 and 32.4% amino acid sequence identity, respectively; Supplementary Fig. 4^14,15^), and the closest homologue in *Arabidopsis thaliana* is the HIPP protein HIPP39 (51.5% amino acid sequence identity between the two HMA-like domains). Nevertheless, using the 3D model of HMA_WTK4_ to assess structural homology using TM-align^51^, we observed that HMA_WTK4_ showed very high (>82%) structural homology to the HMA-like domains of RGA5, Pikp-1 and HIPP39 (Table 1). To extend this analysis, we investigated whether other TKPs also have HMA-like IDs that may have gone unnoticed in previous studies. We found that RPG1, the first TKP identified in barley^52^, also has an HMA-like ID at its N-terminus (52.9% sequence identity with HMA_WTK4_ at the protein level, and 93% at the structural level; Supplementary Fig. 4 and Table 1), as recently described by Reveguk et al.^17^. A recent study described the cloning of *Rmo2* and *Rwt7*, resistance genes against *M. oryzae*, encoding TKPs which also possess HMA-like IDs^53^. None of the other TKPs examined showed any additional domain with structural similarity to HMA_WTK4_ (Supplementary Fig. 5).

**Table 1:** Structure of selected proteins with HMA-like domains and their similarity to HMA_WTK4_

Gene	Species of origin	Resistance	Structure	TM score-HMA_WTK4_^a^
*WTK4*	Wheat	Wheat powdery mildew	TKP	1
*RPG1*	Barley	Barley stem rust	TKP	0.93065
*RGA5*	Rice	Rice blast	NLR	0.83728
*Pikp-1*			NLR	0.82739
*HIPP39*	Arabidopsis	None	HIPP	0.94011

^a^Proteins with a TM score higher than 0.6 (HMA_WTK4_ against the target sequence) are considered structural homologues. A TM score of 1 indicates 100% structural similarity of the two compared 3D structures.

As IDs in NLRs have previously been described to be involved in direct interactions with fungal effectors^10^, we hypothesised that HMA_WTK4_ physically interacts with AvrWTK4^96224^. To test this, we used AlphaFold2 Multimer and AlphaFold3 at three different timepoints to model the complex (detailed description can be found in Supplementary Note). The interface predicted template modelling (ipTM) value, indicative of model confidence, was on average 0.63 for the AvrWTK4^96224^-WTK4 (full-length) in all settings and AlphaFold versions (lowest value: 0.51). For the AvrWTK4^96224^–HMA_WTK4_ interaction, the ipTM value was on average 0.84 (lowest value: 0.71), suggesting a stable association. The model depicted two main interaction surfaces on WTK4: one involving the β2 sheet of HMA_WTK4,_ and one the linker sequence between HMA_WTK4_ and the pseudokinase domain, K1 (amino acids 69–104; Fig. 4B). The main residues predicted to be involved in the interaction surfaces have pLDDT values consistently above 68 for AvrWTK4 and 82 for WTK4, reflecting high confidence in the modelled interface (Supplementary Note). Notably, the T30N substitution found in the virulent isolate THUN-12 and in mutants AvrWTK4_3 and AvrWTK4_6 (Fig. 1C) is located within the predicted surface #2 (Fig. 4B, C). The prediction of the interaction between full-length WTK4 and the virulent Bgt-55150^THUN-12^ variant (hereafter, avrWTK4^THUN-12^) showed variable results depending on the AlphaFold version and parameters used (Supplementary Note). This may suggest a less stable interaction between the virulent avrWTK4 variant and WTK4.

We next validated these interactions *in planta* using a split-luciferase assay in *N. benthamiana*. Our results showed that AvrWTK4^96224^ specifically interacts with HMA_WTK4_ fused to the linker sequence (HMA+linker; Fig. 4D-i). AvrWTK4^96224^ also interacted with full-length WTK4, but to a lesser extent, likely due to its lower protein levels (Fig. 4D-ii and Supplementary Fig. 6). In contrast, the virulent avrWTK4^THUN-12^ variant showed significantly reduced interaction with both constructs, indicating that amino acid residues 30, 33 and 35 in *AvrWTK4* are critical for binding. Protein levels of both effectors were comparable (Supplementary Fig. 6). Both AvrWTK4^96224^ and avrWTK4^THUN-12^ showed very weak interaction with the kinase domains of WTK4 in the absence of HMA_WTK4_ (K1+K2), with luminescence levels similar to the negative control (Fig. 4D-iii). Changing three residues (I29, D30 and Q31) predicted to form the interaction surface #2 in HMA_WTK4_ to three alanine residues (Fig. 4C) led to a significant drop in luminescence for both the full-length WTK4 (WTK4_NoBind_) and the HMA+linker (HMA+linker_NoBind_) constructs, confirming the functional relevance of this interface (Fig. 4D-i, ii). No interaction was detected between WTK4 constructs and BgtE-5764^96224^, the other polymorphic effector from the *AvrWTK4* locus (Fig. 4D).

These results were corroborated by yeast two-hybrid assays: strong interaction was observed between AvrWTK4^96224^ and HMA+linker, while the virulent avrWTK4^THUN-12^ variant showed considerably weaker interaction (-WLH; Fig. 4E). This weak interaction is also consistent with the variable interaction results predicted by AlphaFold (Supplementary Note). The HMA+linker_NoBind_ protein, with a modified Avr binding interface, showed no interaction with AvrWTK4^96224^ (Fig. 4E). No interaction was detected between any WTK4-derived protein and the negative control AvrSr62-7, an unrelated effector recognised by the TKP SR62^TK^^39^. BgtE-5764, the *Bgt* effector used as a negative control in the split luciferase assay, was autoactive in yeast; thus, it could not be used in the yeast two-hybrid assay (Supplementary Fig. 7).

These findings demonstrate that the HMA-like domain of WTK4 mediates direct recognition of AvrWTK4. Alongside insights from the gain-of-virulence *Bgt* mutants, we conclude that virulent *Bgt* isolates evade recognition either through transcriptional downregulation of *AvrWTK4* or via point mutations that specifically affect its HMA-binding interface, reducing direct interaction with the immune sensor. Overall, our results reveal a mechanistic basis for the recognition of effectors by TKPs via integrated decoy domains, similar to ID-containing NLRs. This could lay the groundwork for further exploration of TKP IDs as platforms for engineering immune receptors with broader resistance spectra.

## Discussion

We identified *AvrWTK4*, a powdery mildew effector mediating avirulence on a TKP, using two complementary approaches: bi-parental mapping and mutagenesis. *AvrWTK4* encodes an RNase-like effector, and the pathogen can evade WTK4-mediated recognition by mutating key amino acid residues or by downregulating its gene expression. Importantly, we show that recognition depends on an integrated decoy domain in WTK4 that mediates direct interaction with AvrWTK4.

Strikingly, two independent *AvrWTK4* mutants have the exact same mutation in *Bgt-55150*, resulting in the T30N substitution at the protein level, a mutation that is also present in the naturally virulent isolate THUN-12, suggesting its importance in evasion from recognition (Fig. 4 and Supplementary Note). Other studies, mostly done with NLR immune receptors, have shown that single-point mutations in pathogen effectors allow escape from recognition. For instance, the globally widespread AvrPm8_F43Y variant effectively evades recognition by Pm8^24^.

In addition to amino acid changes, downregulation of expression of *AvrWTK4* represents a second gain-of-virulence mechanism. Our data suggest that mutations resulting in downregulation occur at similar frequencies as amino acid changes: in three out of six mutants, the *AvrWTK4* sequence was unchanged, but its expression was significantly reduced (Fig. 2). In two of these cases (*Bgt* mutants AvrWTK4_1 and AvrWTK4_2), we identified mutations in *Bgt-646*, encoding a deduced ankyrin-repeat-containing protein previously described as a regulator of expression of several effector genes^27^. As described in this earlier work, modifications of *Bgt-646* conferred virulence on both *Pm3a* and *WTK4*, suggesting a shared regulatory pathway^27^. Here, we confirm that *Bgt-646* indirectly determines *WTK4* virulence by controlling *AvrWTK4* expression. In various pathosystems, including *Bgt*, additional genetic components to Avrs, such as suppressors of Avr recognition, have been shown to influence R-Avr interactions^18,26,29,54^. However, in contrast to suppressors that block recognition, *Bgt-646* appears to act as a positive regulator of effector expression. Transcriptional regulators of virulence genes have been described in other fungal species^55^. Whether disruption of regulators of effector gene expressions is a widespread gain-of-virulence mechanism in *Bgt* remains to be elucidated, but it appears to be common at least in the cases of recognition by Pm3a, an NLR^27^ and WTK4, a TKP (this study). Further experiments are needed to elucidate the molecular mechanism by which *Bgt-646* regulates effector gene expression and how many genes are controlled by *Bgt-646*.

Intriguingly, in the case of the mutant AvrWTK4_5, the mutation causing the virulence phenotype remains unknown, as no mutations were found in either *AvrWTK4* or *Bgt-646*. Furthermore, a comprehensive analysis of all sequence polymorphisms in the AvrWTK4_5 mutant failed to identify any mutation in known or predicted genes linked to expression regulation (Supplementary Data 4 and 5). This is consistent with our previous study, in which we could not find the phenotype-causing mutation for three out of seven *Pm3a/WTK4* virulent mutants^27^. This suggests a more complex regulatory network controlling *AvrWTK4* expression and points to additional, yet unidentified, components involved in modulating avirulence on *WTK4*. Future transcriptomic or chromatin-based analyses (e.g. RNA-seq or ChIP-seq) will be needed to determine whether the gain of virulence in these mutants results from indirect regulatory effects or epigenetic changes.

Infections in *Ae. tauschii* lines with a *WTK4*-avirulent *Bgt* isolate triggered a HR (Fig. 3 and Supplementary Fig. 2), and *WTK4*-expressing *Ae. tauschii* protoplasts showed partial but significant cell death upon transfection with *AvrWTK4*^*96224*^ (Fig. 4). This is similar to observations in *WTK3*-containing wheat landraces, where mild cell death occurred after infection with an avirulent *Bgt* isolate^56^. By contrast, *WTK4*-*AvrWTK4*^*96224*^ co-expression failed to induce HR in *N. benthamiana* or wheat (cultivar Fielder) protoplasts, suggesting that *WTK4* requires additional *Ae. tauschii*-specific components. This resembles the Pm4-AvrPm4 interaction, which triggers HR in wheat but not in *N. benthamiana*^26^. Notably, co-expression of WTK4-AvrWTK4 with SR62^NLR^, the helper NLR required for the TKPs SR62^TK^ and RWT4, failed to induce cell death in *N. benthamiana*. This indicates that the WTK4 function does not depend on this known helper and likely requires a distinct one.

In NLR-mediated immunity, sensor NLRs with integrated decoy domains often require helper NLRs to fully activate defence responses, as described for RGA5/RGA4 in rice or RPS4/RRS1 in *Arabidopsis*^14,57–59^. Likewise, TKPs such as SR62^TK^ and RWT4 were shown to require a helper NLR to mediate resistance to stem rust and wheat blast, respectively^39,40^. Similarly, in the case of the tandem kinase gene *Yr15*, different introgression lines showed varying resistance phenotypes, indicating that *Yr15* is part of a larger resistance complex involving additional genetic components^60,61^. Given that WTK4 has an ID that directly binds to the AvrWTK4 effector, we hypothesise that it might act as a sensor, for which the putative helper NLR is not yet known, and whose presence would trigger HR in *WTK4-AvrWTK4* co-expression assays. The fact that HR could be observed in a susceptible *Ae. tauschii* line from lineage 2 upon co-transfection of *WTK4* and *AvrWTK4* (Fig. 3), but not in wheat cultivar Fielder (Supplementary Fig. 3), indicates that the putative helper of WTK4 is private to *Ae. tauschii*. TKPs and their NLR partners are frequently genetically linked and co-evolve, so they can be lost individually or together, with a consequent loss of resistance, even within the same species or lineage^62^. However, to determine whether the putative helper of WTK4 is lineage- or species-specific, additional experiments involving *Ae. tauschii* lines outside lineage 2 would be required. Our data, together with the existing literature, support a broader model in which TKPs act as sensors that depend on helper proteins (possibly NLRs) for full immune activation. Alternatively, it is also possible that AvrWTK4 manipulates WTK4 in a way that is sensed by a putative 'guard' protein, which is required to trigger downstream signalling and the immune response. To find the additional component required for *WTK4* function, interaction screens, such as yeast-two-hybrid or immunoprecipitation followed by mass spectrometry (IP-MS), or extensive mutant analyses in search of second-site mutations, may be performed.

The discovery of an HMA-like ID in WTK4 provides a mechanistic basis for studying resistance conferred by TKPs. A recent study found that over half of TKPs across the plant kingdom carry IDs^17^. Moreover, the presence of IDs in at least six characterised resistance proteins with a TKP structure (WTK4, RPG1, Lr9, RWT4, Rmo2 and Rwt7; this study^38,53,63^), suggests a common, functional role in pathogen recognition. However, the specific contributions of these domains remain largely unexplored. In the case of RWT4, KDup is directly involved in the interaction with the *M. oryzae* effector AvrPWT4^38^. Lr9 has an additional vWA domain, but to our knowledge, this ID has not yet been functionally characterised^63^. Our findings reinforce the emerging view that IDs within TKPs may play central roles in effector recognition.

Several effectors known to interact with integrated HMA-like domains of NLRs, such as AVR-Pia, AVR1-CO39 and AVR-Pik, belong to the MAX (*Magnaporthe* Avrs and ToxB-like) effector family, a structurally conserved group common in *M. oryzae*^14,15,64^. *WTK4* confers resistance against *B. graminis* f. sp. *tritici*, a pathogen which lacks MAX-like effectors in its repertoire^64,65^. Its corresponding effector, AvrWTK4, belongs to the RNase-like family and has no sequence or structural similarity with MAX effectors^29,65^. This shows that effectors from different pathogen species have convergently evolved to target and bind HMA domains from their hosts. This convergence, where distinct effector families target HMA-like domains, suggests that these domains are 'hotspots' for pathogen manipulation. Furthermore, HMA-like IDs appear capable of evolving diverse effector-binding specificities, possibly through minimal sequence changes. Unlike NLR HMA-like IDs, which co-evolve with MAX effectors^64^, the interaction between HMA_WTK4_ and the RNase-like AvrWTK4 effector likely reflects an independent adaptation to the powdery mildew pathogen.

For future applications, it will be important to determine whether different effector types bind overlapping or distinct surfaces on HMA domains. AVR1-CO39 binds HMA_RGA5_ primarily through its α1/β2 surface. In contrast, AVR-PikD binds HMA_Pikp-1_ via its β2/β3/β4 surfaces. These distinct binding interfaces have been successfully combined in an engineered HMA-like domain to confer dual recognition specificity^14,15,66^. The *M. oryzae* effector Pwl2 binds HMA_OsHIPP43_ through an extensive interface involving mainly α1, β3 and the loop between α1 and β2, and this knowledge has also been used to design a synthetic receptor with broad-spectrum resistance^16^. A recent study presented the targeted engineering of the HMA-like domain of the TKP Rmo2 to recognise the wheat blast effector PWT7, in addition to the naturally recognised PBY2, highlighting the potential of this approach in TKPs^67^. In this study we predicted, and then experimentally showed, that AvrWTK4 binds to HMA_WTK4_ primarily through its β2 surface, and that modifications of three residues in the β2 surface is enough to disrupt AvrWTK4 binding (Fig. 4). Such knowledge could potentially be used to design synthetic HMA modules capable of recognising a broader range of Avr effectors, even from unrelated pathogens, such as *Bgt* and *M. oryzae*. In parallel, nanobody-based IDs have been introduced into NLRs to expand recognition potential, offering another promising approach for engineering immune receptors^68^. These studies show that the identification and study of IDs in plant immune receptors are of high relevance, illustrating their potential as platforms for designing immune receptors with broadened or redirected specificity.

In conclusion, we demonstrated that the identification of Avr effectors is instrumental to understand resistance mechanisms of nonconventional immune receptors. Identifying and characterising *AvrWTK4* provided valuable insights into race-specific resistance mediated by TKPs. We showed that an integrated HMA-like domain in WTK4 recognises AvrWTK4, and that *Bgt* can evade this recognition through point mutations or effector downregulation. These findings further highlight the versatility of HMA-like IDs in pathogen sensing and underscore the importance of combining genetic, molecular, and structural approaches to unravel ETI. From a breeding perspective, leveraging such mechanistic insights enables more informed deployment of immune receptors and opens the door to engineering broad-spectrum resistance in cereal crops through rational design of TKPs with optimised IDs.

## Methods

### Fungal and plant material

All *Bgt* mutants described in this study were derived from the isolate CHE_96224^44^ and generated as previously described (see also Methods section 'Mutagenesis and phenotyping experiments')^27^. The F1 mapping population derived from the crossing of CHE_96224 and THUN-12, used for mapping the *AvrWTK4* locus, was also previously described^44^.

To maintain the *Bgt* isolates, the powdery mildew-susceptible wheat cultivars Kanzler (accession number K-57220) and Chancellor (accession number K-51404) were used. *Aegilops tauschii* lines (i.e. TOWWC0087, TOWWC0112 and TOWWC0154) from a diversity panel^43,69^ containing the resistance gene *WTK4* were used to assess powdery mildew virulence on *WTK4*. For evaluating the virulence specificity of the *Bgt* mutants we used the wheat lines Axminster/8*Chancellor (*Pm1a*^70^), Federation*4/Ulka (*Pm2a*^71^*)*, Asosan/8*Chancellor (*Pm3a*), Chul/8*Chancellor (*Pm3b*), Sonora/8*Chancellor (*Pm3c*), Kolibri (*Pm3d*), W150 (*Pm3e*), Michigan Amber/8*Chancellor (*Pm3f*^72^), Khapli/8*Chancellor//8*Federation (*Pm4a*), Federation*8/W804 (*Pm4b*^48^*)*, Kavkaz/4*Federation (*Pm8*^73^), Amigo (1AL.1RS translocation line containing *Pm17*^25^) and the *Pm24*-containing line (kindly provided by C. Cowger).

### QTL mapping

Selected F1 progeny of the CHE_96224 x THUN-12 cross^44^ were phenotyped on the *Ae. tauschii* lines TOWWC0028, TOWWC0040 and TOWWC0111^43,69^ and scored at 7–8 days post-infection (Supplementary Data 2). The cultivar Kanzler was used as a wheat powdery mildew susceptible control. The phenotype was scored for individual leaf segments with a value between 0 (completely avirulent) and 100 (completely virulent), corresponding to the percentage of the leaf covered by fungal mycelium. To ensure consistency, all leaf area coverage scores were evaluated by a single trained investigator based on at least three independent biological replicates. The final score used for QTL mapping consists of an average of two to four leaf segments per *Ae. tauschii* line. QTL mapping was performed with a single interval approach using the R/qtl v.1.46.2 (https://rqtl.org/) package in R/RStudio (versions 4.3.2 and 2023.09.1, respectively). The commands used were read.cross(), jittermap() and calc.genoprobe(step = 1), as described previously^44^. Single interval analysis was performed using the scanone(model = 'np') method. LOD thresholds were calculated using 1000 permutations.

### Mutagenesis and phenotyping experiments

*Ae. tauschii* and wheat plants used for mutagenesis and phenotyping experiments were grown for 12–17 days (*Ae. tauschii*) or 9–12 days (wheat) under the following conditions: 16 h light, 8 h dark, 18 °C, and 60% humidity. First leaves were then cut into 3-cm fragments and placed in Petri dishes containing water agar supplemented with benzimidazole (5 ppm, MERCK, 51-17-2). Two to four biological replicates were then spray-inoculated with *Bgt* isolates as described in our previous study^27^.

To identify *Bgt* mutants with a gain of virulence on *WTK4*, we used the AvrXpose approach^27^. Briefly, the *Bgt* isolate CHE_96224 was grown on the susceptible wheat line Kanzler and irradiated with UV-C light three times. The irradiated spore mixture was then used to inoculate *Ae. tauschii* lines containing *WTK4* (TOWWC0087, TOWWC0112 and TOWWC0154). Surviving *Bgt* colonies on either one of these lines were single-spore isolated and propagated on Kanzler until sufficient spores were available for further phenotyping and DNA extraction. The phenotype of the *AvrWTK4* mutants was further evaluated on different *Pm* gene-containing lines to confirm their virulence specificity to *WTK4*. Disease severity was assessed by eye 7–8 days after inoculation for at least three biological replicates per line, and the virulence was based on the percentage of the leaf area covered by the fungal mycelium.

### DNA extraction and whole genome sequencing

The progeny of the *Bgt* F1 mapping population had already been sequenced, and a linkage map was available^44^. For the newly identified *Bgt* mutants, DNA was extracted using a chloroform- and CTAB-based protocol as previously described^18^. Library preparation and Illumina whole-genome sequencing were performed at Novogene (Cambridge, UK) using the NovaSeq 6000 technology, as described in our previous study^27^. A minimum of 12 Gb of sequence data (average coverage: 100×) was generated per *Bgt* isolate, with paired-end sequence reads of 150 base pairs (bp) and an insert size of approximately 350 bp.

### Trimming, mapping, variant calling and transposable element detection

To prepare the Illumina reads for subsequent analysis, raw reads were first trimmed to remove Illumina oligo adapters, and sequence quality was assessed using Trimmomatic v. 0.39^74^. Trimmed reads were then mapped to the reference genome of the *Bgt* isolate CHE_96224, genome version 3.16^44^, using bwa mem v0.7^75^. We then sorted, removed duplicates, generated, and indexed the mapping (bam) files using SAMtools^76,77^.

FreeBayes was used to perform the haplotype calling, applying the parameters -p 1 -m 30 -q 20 -z 0.03 -F 0.7 -3 200^78^. The TASSEL5 software was used to convert the resulting vcf file into HapMap format^79^. The software Detettore was used to detect TE insertions^80^. An in-house Python script was used to detect duplicated or deleted genes (https://gist.github.com/caldetas/24576da33d1ff91057ecabb1c5a3b6af). Variants located within 1.5 or 2 kb windows from annotated genes (for SNPs and TE insertions, respectively) were selected and visually inspected using the integrative genomics viewer software IGV^81^.

### PCR, qPCR experiments and statistical analyses

The PCR amplification of *Bgt-55150* in the *AvrWTK4* mutants was performed using the Phusion® High-Fidelity DNA Polymerase (M0530S, New England BioLabs Inc.) following the manufacturer’s recommendations, with an annealing temperature of 59 °C and an extension time of 45 s. The sequences of the primers used in all PCR and RT-qPCR experiments are described in Supplementary Data 6.

RT-qPCR experiments to study the expression of *Bgt-55150* and *Bgt-646* were performed according to the MIQE guidelines^82^, following the procedure described in our previous study^27^. Briefly, the Dynabeads mRNA DIRECT Kit (Invitrogen) was used to extract mRNA from infected leaf material obtained from three to five independent biological replicates, 2 days after *Bgt* inoculation. cDNA was synthesised using the Maxima H Minus First Strand cDNA Synthesis Kit (ThermoFisher), according to the manufacturer’s instructions. The KAPA SYBR FAST qPCR kit (Kapa Biosystems) was used for the reaction. Expression was then measured in a CFX384 Touch Real-Time PCR Detection System (Bio-Rad). Gene expression was analysed using CFX Maestro software version 3.1 (Bio-Rad), and fungal glyceraldehyde 3-phosphate dehydrogenase (*GAPDH*_*Bgt*_) expression was used to normalise gene expression. An analysis of variance (ANOVA) followed by a Tukey HSD test was conducted to assess statistical differences between CHE_96224 and the *Bgt* mutants (Supplementary Data 7). All analyses were performed in R using the aov and TukeyHSD functions, respectively. Finally, the glht and cld functions of the multcomp package were used to assign significance groups^83^.

### Microscopic evaluation of the immune response in *WTK4* containing *Ae. tauschii* lines

Twelve-day-old leaves of *Ae. tauschii* lines TOWWC0028 and TOWWC0077 (containing *WTK4*) were cut into 5 cm fragments, placed on water agar petri dishes, and infected at low density with the *Bgt* isolate CHE_96224. At 24, 48 and 96 h post-infection (hpi), two leaves per line were destained with 3:1 ethanol:glacial acetic acid, then stained first overnight with a DAB solution (5 mM 3'3-diaminobenzidine, pH 3.8) to visualise cell death, and then for 1 min with a Coomassie blue solution to visualise the fungal structures (0.15% Coomassie blue R-250 in methanol). The leaves were then rinsed twice with distilled water and stored in lactoglycerol (equal parts lactic acid, glycerol and water) until microscopy. At least 100 randomly selected spores per line and time point were categorised according to the outcome of the cell attack attempt: (i) penetration attempt with consequent HR, (ii) penetration attempt blocked by the formation of papilla, (iii) penetration and appressorium formation and (iv) penetration and microcolony formation. Examples of these reactions can be found in Supplementary Fig. 3. If none of these reactions occurred, or if it was not clear which reaction had occurred, the spores were assigned to the category (v), unclear reaction/no penetration.

### Cloning of expression constructs

The coding sequences of *Bgt-55150*_*96224*_*, Bgt-55150*_*THUN-12*_*, BgtE-5764*_*96224*_ and *BgtE-5764*_*THUN-12*_ without signal peptides (predicted by SignalP6.0; https://services.healthtech.dtu.dk/services/SignalP-6.0/) were codon-optimised for *N. benthamiana* expression with the codon optimisation tool from Integrated DNA technologies (https://eu.idtdna.com) and synthesised with Gateway-compatible attL sites by BioCat GmbH (https://www.biocat.com). Sequence information of all constructs produced by gene synthesis can be found in Supplementary Data 8. The *WTK4* coding sequence was obtained from *Ae. tauschii* cDNA and can be retrieved from NCBI (GenBank: MW295405.1). Entry clones described above were cloned into the binary expression vectors pIPKb002, pIPKb004^84^, and the pDEST vectors for the split luciferase assay (GW_NLUC, NLUC_GW, GW_CLUC and CLUC_GW^85^) using the Gateway LR Clonase II (Invitrogen). The constructs were subsequently transformed into *Agrobacterium tumefaciens* strain GV3101 using a freeze-thaw transformation protocol^86^.

### Cell death and interaction assays in *N. benthamiana* and wheat protoplasts

*A. tumefaciens-*mediated expression of resistance and effector genes was achieved following the procedure described previously^46^. For the cell death assay, *A. tumefaciens* carrying the effector or *R* gene in pIPKb004^84^ were mixed in a ratio of 2:1, with the final addition of 10% volume of the P19 *A. tumefaciens* strain immediately prior to infiltration. HR development was assessed 4–6 days after infiltration using a Fusion FX imaging System (Vilber Lourmat, Eberhardzell, Germany). The cell death experiment was repeated three times, and a representative image was chosen for the results (Supplementary Fig. 4). For the split luciferase assay, the effector, the *R* gene, and the P19 *A. tumefaciens* strain were mixed in equal proportions immediately prior to infiltration. Three days after infiltration, 2 leaf discs (technical replicates) from 6 to 10 biological replicates were cut, a D-luciferin substrate was added, and luminescence was measured continuously for 25 min at 200 ms/well using a Tecan Infinite 200 PRO LUMI plate reader.

To test cell death in wheat protoplasts, a previously described protocol was used^49^. Briefly, plasmid DNA of *R* and *Avr* genes, previously cloned into pTA22^49^, was extracted using the Xtra Midi Plus Endotoxin-free MidiPrep Kit from Macherey-Nagel. Seedlings of the *Ae. tauschii* lines TOWWC0111 and TOWWC0167 and of the wheat cultivar Fielder were grown in a growth chamber under a cycle of 12 h light (100 µmol m^−2^ s^−1^) and 12 h dark at 24 °C for 10–12 days (*Ae. tauschii*) or 7–8 days (wheat). Cell walls of epidermal peels were digested in a cellulase RS and macerozyme R-10 (Onozuka) solution, then filtered through a 40 μm nylon cell strainer and centrifuged for 3 min at 80 × *g*. The cells were then resuspended in W5 solution (2 mM MES-KOH pH 5.7, 5 mM KCl, 125 mM CaCl_2_, 154 mM NaCl), incubated on ice, then resuspended in an appropriate volume of MMG solution (4 mM MES-KOH pH 5.7, 0.4 M mannitol, 15 mM MgCl_2_) to obtain 2–3 × 10^5^ cells per ml. Then, a DNA solution containing 3 pmol of each construct and 3 pmol of a reporter plasmid containing *YFP* was prepared. The DNA solution was then mixed in a 2 mL tube with 200 μL of the protoplast solution and ~230 μL of the PEG solution (40% w/v PEG-4000, 0.2 M mannitol, 100 mM CaCl_2_), gently homogenised by slowly inverting the tube, and incubated for 15–30 min at room temperature. Finally, 920 μL of W5 solution were added to stop the transformation reaction. Transformed protoplasts were then centrifuged at 100 × *g* for 2 min, resuspended in 650 μL of W5 solution, transferred to 12-well cell culture plates, and incubated at 23 °C for 16–20 h in the dark. After incubation, 350 µL were removed, leaving the remaining protoplasts in 300 µL W5 solution. YFP fluorescence was measured in two technical replicates and three biological replicates per treatment using a plate reader. Within each experiment, the obtained values were normalised (i) to the median value of the GUS (TOWWC0111) or WTK4 + GUS (TOWWC0167) treatment, and (ii) to the median value of a treatment set (i.e. values of biological replicate 'a' for all treatments represent the treatment set 'a'). Measurements outside of the median ± 2 × interquartile range were considered as outliers and removed from the analysis. The experiment was repeated three times for TOWWC0111 (nine biological replicates in total), and four times for TOWWC0167 (twelve biological replicates in total). A one-way ANOVA followed by multiple comparisons of means (contrast analysis) was used to estimate significant differences between treatments.

### Yeast two-hybrid assay

Yeast two-hybrid assays were conducted using the MATCHMAKER GAL4 system (Clontech, USA) following the manufacturer’s instructions. The coding sequences (CDS) of full-length and truncated forms of *WTK4* were cloned into the pGADT7 vector (Clontech, USA, PT3249-5) using the *EcoRI* and *BamHI* restriction sites. The CDS of the effector genes were cloned into the pGBKT7 vector (Clontech, USA, PT3248-5) using the same restriction sites. The resulting AD and BD fusion constructs were co-transformed into *Saccharomyces cerevisiae* strain AH109 and plated on synthetic dropout (SD) medium lacking tryptophan and leucine (SD-WL) for selection. Three individual colonies from SD-WL plates were resuspended in sterile water, adjusted to an optical density at 600 nm (OD_600_) of 1.0, and subsequently diluted 1:10 and 1:100. Aliquots of each dilution were plated onto SD-WL and selective SD medium lacking tryptophan, leucine and histidine (SD-WLH), and incubated for 3–5 days at 30 °C to assess protein–protein interactions.

For protein detection, two to three individual yeast colonies were inoculated into 1 mL of SD-2 liquid medium in 2 mL centrifuge tubes and incubated overnight at 30 °C with shaking at 220 rpm. The cultures were centrifuged at 1000 × *g* for 5 min to pellet the cells. After discarding the supernatant, cell pellets were resuspended in 1 mL of 0.2 M sodium hydroxide and incubated at room temperature for 5 min. Cells were then pelleted again by centrifugation at 1000 × *g* for 5 min, the supernatant removed, and the pellets resuspended in 100 μL of protein loading buffer along with glass beads. The samples were vortexed for 5 min to lyse the cells, followed by heating at 95 °C for 10 min. The resulting protein extracts were subsequently used for immunoblotting analysis.

### Protein detection for split-luciferase and cell death assays

The soluble protein fraction was extracted from *N. benthamiana* leaves 3 days after infiltration with *A. tumefaciens*. Six leaf discs in total (0.5 mm diameter) from 3 infiltrated leaves were pooled, ground in liquid nitrogen, and resuspended in 2× modified Laemmli buffer (100 mM Tris-HCl pH 6.8, 200 mM DTT, 0.04% bromophenol blue, 20% glycerol, 2% SDS), boiled at 90 °C for 5 min, and centrifuged at 17,000 × *g* for 1 min. Twenty microlitres of total protein extract were separated in SDS polyacrylamide (PA) gels (7.5%, 10% or 12%) and semi-dry blotted to a Nitrocellulose membrane using a Trans-Blot SD Semi-Dry Transfer Cell (Bio-Rad). For LUC tag detection, the primary antibody (anti-LUC, rabbit polyclonal, Sigma-Aldrich) was used at a dilution of 1:3000, membranes washed with TBST and then incubated with anti-rabbit peroxidase antibody (anti-rabbit-HRP, mouse, LabForce) diluted 1:3000. Peroxidase chemiluminescence was detected using a Fusion FX Imaging System (Vilber Lourmat, Eberhardzell, Germany) after application of a WesternBright ECL HRP substrate (Advansta). The membranes were also incubated with Ponceau stain for total protein detection.

### Structural modelling and other bioinformatic analyses of HMA_WTK4_

The 3D modelling of AvrWTK4 and WTK4 was performed using AlphaFold2 and AlphaFold3^50,87^, and the interaction between AvrWTK4 and WTK4 using AlphaFold multimer through ColabFold^50^ or the AlphaFold server (https://alphafoldserver.com/). Details on the AvrWTK4–WTK4 interaction modelling can be found in the Supplementary Note.

The protein sequences from TKPs RPG1, YR15, SR60 and SR62^TK^ have been retrieved from UniProt (https://www.uniprot.org/). HIPP39 was found through blastp of HMA_WTK4_ with standard parameters in the NCBI database (https://blast.ncbi.nlm.nih.gov/). Their 3D structures were predicted with AlphaFold2 and visualised using PyMOL. The HMA-like domain of WTK4 was annotated following the ProSite automated annotation, and the HMA-like domain of RPG1 was annotated using the multiple sequence alignment of the HMA domains of known R proteins and HMA_WTK4_, shown in Supplementary Fig. 4 (performed using the Clustal Omega software; https://www.ebi.ac.uk/jdispatcher/msa/clustalo). TM-align was used to infer structural similarity between HMA_WTK4_ and RPG1, RGA5, Pikp1 and HIPP39, using their PDB structure files as input^51^. Any structure with a TM score greater than 0.6, indicating a 60% structural similarity, was considered a structural homologue.

### Reporting summary

Further information on research design is available in the Nature Portfolio Reporting Summary linked to this article.

## Supplementary information

Supplementary Information

Descriptions of Additional Supplementary Files

Supplementary Data 1

Supplementary Data 2

Supplementary Data 3

Supplementary Data 4

Supplementary Data 5

Supplementary Data 6

Supplementary Data 7

Supplementary Data 8

Reporting Summary

Transparent Peer Review File

## Source data

Source data

## Data Availability

Raw FASTA sequences of all Bgt mutants generated in this study have been deposited in the NCBI database under BioProject accession code PRJNA1016363. The sequences of the constructs used in this study are provided in the Supplementary Data. Source data are provided with this paper.
