# Supplementary material for: An HMA-like integrated domain in the wheat tandem kinase WTK4 recognises an RNase-like pathogen effector
Source: Nat Commun. 2026 Jul 6;17:8458. doi: 10.1038/s41467-026-74940-x (PMC13478195; doi:10.1038/s41467-026-74940-x)
Supplement: Supplementary file 2 [file 41467_2026_74940_MOESM12_ESM.pdf]

nature portfolio

Peer Review File

An HMA-like integrated domain in the wheat tandem kinase WTK4 recognises an RNase-like pathogen effector

Corresponding Author: Dr Zoe Bernasconi

This file contains all reviewer reports in order by version, followed by all author rebuttals in order by version.

Version 0:

Reviewer comments:

Reviewer #1

(Remarks to the Author)
This manuscript identifies and characterizes AvrWTK4, the effector from wheat powdery mildew recognized by the tandem kinase protein (TKP) immune receptor WTK4. The authors use a combination of genetic mapping, mutagenesis, biochemical interaction assays, and structural modeling approaches. The discovery of AvrWTK4 and the mechanistic characterization of its interaction with the HMA-like domain of WTK4 is a substantial contribution to the field of plant immunity. The work is timely, novel, and provides a clear mechanistic model for how TKPs can recognize effectors via integrated decoy domains, paralleling the established mechanism in NLRs. The experiments are well-designed, the data are robust, and the conclusions are supported by the evidence.

Comments & Suggestions:

1. The current title can be revised more directly to state the core mechanistic finding. For example: "A wheat tandem kinase immune receptor recognises a powdery mildew effector via an integrated HMA-like decoy domain", or "Direct effector recognition by a tandem kinase protein is mediated by an integrated HMA-like domain"

2. Adjust the abstract's final sentence from "These findings further highlight..." to a stronger statement like: "We establish that an integrated HMA-like domain serves as the direct effector-binding decoy for WTK4, providing a mechanistic paradigm for effector recognition by tandem kinase immune receptors."

3. Line 79: When mentioning Bgt Avrs (e.g., AvrPm2, AvrPm3), add a brief note that these are NLR-recognized to contrast with the TKP-recognized AvrWTK4 (the manuscript's focus).

4. Line 167-168: The exception of AvrWTK4_5 being virulent on Pm3f is noted but not discussed. A brief sentence explaining that this is likely due to an unrelated, secondary mutation would be helpful.

5. Line 206-208: Address “unknown mutation” in AvrWTK4_5: The manuscript notes AvrWTK4_5 has reduced AvrWTK4 expression but no mutations in Bgt-646/AvrWTK4. Add a brief statement about exploratory analyses (e.g., “We screened for mutations in 10 known effector regulators (e.g., XYZ; Bernasconi et al. 2024) but found none; future RNA-seq or ChIP-seq will test for indirect regulatory cascades”). This avoids leaving the gap unaddressed.

6. The current data showing that cell death requires an Ae. tauschii-specific component (Fig. 3, Supp. Fig. S3) is intriguing but currently feels like an unresolved thread. Co-expression of Ae. tauschii-derived helper NLR candidates (e.g., orthologs of WTK3 and Sr62’s helper NLR WTN1/Sr62-NLR; Lu et al. 2025; Chen et al. 2025) may be texted in N. benthamiana. Even negative data will strengthen the conclusion that WTK4 requires a co-factor that is not a common canonical helper NLR.” The discussion (lines 415-424) correctly links this to the emerging theme of helper NLRs for TKPs. In the Results section (line 261-265), after presenting the negative data in N. benthamiana and wheat, try to add a sentence like: "This suggests that WTK4-mediated cell death requires an additional host-specific factor, consistent with recent models where TKPs act as sensors that rely on helper proteins for full immune activation (Chen et al., 2025; Lu et al., 2025)." This frames the finding as part of an active area of research rather than a limitation.

7. In the text (lines 306-308), explicitly state what the "linker sequence" is (e.g., "the ~XX amino acid segment connecting the

HMA domain to the K1 pseudokinase domain").

8. Line 372: "It is noteworthy that" –> This is a slightly weak transition. Consider "Strikingly," or "A key finding is that".

9. Line 425: "represents a significant advance" –> This is an opinion. It's better to let the data speak for itself. Consider rephrasing to: "The discovery of an HMA-like ID in WTK4 provides a mechanistic basis for..."

10. Line 439: “MAX-like effectors in its repertoire”. Specify “B. graminis f. sp. tritici” instead of “its.”

11. In the discussion (around lines 443-447), briefly speculate on how the HMA domain of WTK4 evolved to recognize this specific RNase-like effector, unlike the HMA domains in rice that recognize MAX effectors. Is the binding surface fundamentally different? This reinforces the potential for engineering and sets the stage for future work.

12. Clarify phenotyping details: In bi-parental mapping, specify how the 0–100 virulence score was calibrated (e.g., “% leaf area covered by mycelium, with three technical replicates per leaf segment”) and whether inter-observer consistency was assessed. This strengthens the QTL mapping validity.

13. Validate structural model confidence: The AlphaFold Multimer results (ipTM 0.73–0.86) are strong, but add pLDDT scores for the interaction interface residues (e.g., “Residues I29/D30/Q31 in HMAWTK4 and T30 in AvrWTK496224 had pLDDT > 90, indicating high structural confidence”). This supports the functional relevance of the predicted interface.

14. Expand on the finding that AvrWTK4 (RNase-like) binds HMAWTK4, whereas NLR HMA domains bind MAX effectors: “This convergence—distinct effector families targeting HMA domains—suggests HMA-like IDs are ‘hotspots’ for pathogen manipulation. Unlike NLR HMA domains, which co-evolve with MAX effectors (Seong & Krasileva 2021), HMAWTK4’s interaction with RNase-like AvrWTK4 may reflect independent adaptation to powdery mildew.”

15. Supp. Fig. S1: Add Virulent (+)/Avirulent (-) entries for each isolate-R gene combination in the Table.

Reviewer #2

(Remarks to the Author)
The manuscript by Zoe Bernasconi et al. reports the isolation of the avirulence effector AvrWTK4 from the wheat powdery mildew pathogenic fungus, which is recognized by the wheat tandem kinase (TKP) ‚immune sensor’ WTK4. The authors identified AvrWTK4 through the use of two independent methods, a bi-parental powdery mildew mapping population and a UV mutagenesis-based approach and show that the gene encodes a deduced RNase-like protein (RALPH). They also present evidence that another powdery mildew gene, Bgt-646, encoding a predicted ankyrin-repeat containing protein, is required for AvrWTK4 expression and thus mutants in Bgt-646 render an AvrWTK4 containing isolate virulent on WTK4 containing wheat. They then present evidence that co-expression of AvrWTK4 with WTK4 induces a mild cell death phenotype only in Ae. tauschii protoplasts. AlphaFold-based structural models suggests that AvrWTK496224 interacts with a previously unnoticed HMA domain at the N-terminus of WTK4 while the virulent variant of AvrWTK496224 (avrWTK4THUN-12) does not. Limited experimental support for this is provided by split-luciferase experiments in planta and yeast two-hybrid experiments. These experiments show that AvrWTK496224 interacts with the HMA+linker region, while a much weaker interaction with full-length WTK4 is observed.

Overall, the study presents an important advance in understanding the function of a TKP in grasses in an isolate-specific plant immune response. Text and Figures are well organized and many of the claims of the manuscript are supported by experimental evidence.

I have the following major comments

1. A key observation by the authors is that co-expression of AvrWTK4 with WTK4 induces a mild cell death in Ae. tauschii protoplasts (33% reduction in relative fluorescence intensity; Fig. 3D and 3E), but not in N. benthamiana and hexaploid wheat (cv. Fielder). Given the mild cell death phenotype detected, it will be important to substantiate the specificity of the effector-triggered and WTK4-mediated (and indirectly inferred) cell death using the virulent version AvrWTK4-3 (or AvrWTK4-6 leading to a T30N substitution in the deduced protein) described in this study. It will also be important to extend these experiments to a known Bgt RALPH effector belonging to the same RNase-like subfamily as AvrWTK4 and showing the greatest sequence similarity to this avirulence effector.
2. In the discussion the authors conclude that ’together, these findings support a broader model in which TKPs act as sensors that depend on helper proteins (possibly NLRs) for full immune activation’ (ln 420-422). An alternative model is that WTK4 is not an immune sensor, but instead a putative Ae. tauschii lineage-specific sensor NLR indirectly detects manipulation of WTK4 by AvrWTK4. I mention such an alternative model also because the data claiming a direct interaction between AvrWTK4 and full-length WTK4 are not conclusively convincing (e.g. undetectable in yeast two-hybrid experiments; Fig. 4E). If the helper NLR model proposed by the authors is correct, it would be expected that protoplasts of phylogenetically divergent Ae. tauschii genotypes would all be capable of mediating cell death upon co-expression of WTK4 and AvrWTK4, since helper NLRs are typically conserved in a phylogenetic plant lineage. This will also clarify whether the incidence of effector-triggered and WTK4-mediated cell death is quantitatively similar or dissimilar in phylogenetically distant Ae. tauschii lines. These additional experiments can be conducted in a reasonable time frame and the discussion need to be modified accordingly. Based on the data presented, the authors cannot claim that WTK4 is a ‘wheat tandem

kinase immune receptor' (lines 39–40 in the abstract and throughout the manuscript).
3. In Line 264-266, the authors state that "These findings indicate that at least one genetic component unique to Ae. tauschii and absent in N. benthamiana or the wheat cultivar Fielder is needed for inducing HR upon recognition of Bgt-5515096224." To substantiate this statement, protein detection should be performed for the N. benthamiana cell death assay, as the absence of cell death upon co-expression of WTK4 and AvrWTK4 may also be explained by low accumulation of one of the two proteins. Additionally, whereas the protoplast assays performed in cv. Fielder contain the appropriate controls, the protoplast assays shown in Figure 3D should also contain at least one positive control (e.g the Sr50-AvrSr50 pair).
4. In line 318 the authors state that "AvrWTK496224 also interacted with full-length WTK4, but to a lesser extent, likely due to its lower protein levels (Fig. 4D ii, Supp. Fig. S7)." The samples need to run on the same gel/blot in order to compare protein levels between AvrWTK496224 and WTK4. In addition, the size marker needs to be shown, control lane needs to be included and full-length blots need to be provided. This experimental setup should be considered for Supp. Fig. S8 as well.
5. In line 353 the authors state "Residues involved in interactions are highlighted in grey and underscored." This needs to be corrected to "…predicted to be involved in interactions…". Only a high-resolution structure can provide conclusive evidence for this claim.
6. In line 372 "Structural modelling and interaction assays confirmed that this mutation strongly reduces interaction with the HMA domain of WTK4". This statement must be toned down for the following reasons: when using AlphaFold3 (AF3) (Template setting cutoff date 3/2/2025), the interaction between the deduced WTK HMA domain and AvrWTK4(Thun12) produces a higher ipTM score compared to the interaction with AvrWTK496224. See the interactions predicted below:

HMA-linker + AvrWTK4Thun12 : ipTM = 0.82 pTM = 0.77
HMA-linker + AvrWTK496224 : ipTM = 0.81 pTM = 0.77
HMA + AvrWTK4Thun12 : ipTM = 0.9 pTM = 0.87
HMA + AvrWTK496224 : ipTM = 0.89 pTM = 0.87
HMARPG1(=1-73) + AvrWTK4Thun12 : ipTM = 0.89 pTM = 0.88

Thus, the structural modelling does not "confirm" this specific interaction. Similarly, in 326 the authors state "…confirming the functional relevance of this interface (Fig. 4D i-ii)." This needs to be changed to something along the lines "… providing evidence that suggests the predicted interface is functionally relevant…".

Moreover, in AF3, prediction of the complex between Full-length (FL) WTK4 and AvrWTK4 is consistently poor (ipTM 0.17; Template setting cutoff date 2/3/2025). Thus, it is necessary to describe the exact parameters used to obtain the predicted complex between FL WTK and AvrWTK4, as shown in main Figure 4. Are the predictions with FL WTK reproducibly higher than >0.6 ipTM with the parameters used by the authors?
7. Line 242-245 (Fig. 3A-B): "These data provide evidence that the WTK4-induced immune response results in both HR and papilla formation, very similar to other Pm resistance genes such as Pm4, encoding a kinase fusion protein (Sánchez-Martín et al. 2021)." I doubt that papilla formation is a defence response specifically to WTK4-containing lines (or other Pm gene-mediated resistant wheat lines), and the data provided do not permit this conclusion. The data shown in Fig 3A and 3B only show individual plant-pathogen interaction sites and do not allow quantitative conclusions to be drawn. It is well documented that the formation of papillae in response to powdery mildew inoculation is commonly observed in virulent powdery mildew-host interactions.

Minor points:

1. Ln 375: please tone down the statement that the apparent reduction in binding is "strong" or provide quantitative interaction assays (e.g. ITC). Western blotting is sufficient to claim that the effector protein levels are comparable, but quantitative differences in protein levels may contribute to the reduction in cell death activity.
2. Line 194: "encoding an ankyrin-repeat-containing protein". This needs to be corrected to "encoding a deduced ankyrin-containing protein" throughout the manuscript.
3. It is a common misconception that the split-luciferase assay detects protein-protein interactions in vivo, as the analysis involves tissue destruction and it remains unclear whether the protein interactions occur only after cell homogenization. This must be changed in line 315 and throughout the manuscript.
4. The reference list is in parts outdated and lacks a number of recent publications describing high resolution structures of RNase-like powdery mildew avirulence effectors and the structure of a heterocomplex of such an avirulence effector with its matching sensor NLR. These are relevant for a more general interpretation and discussion of the diversity of RNase-like effector interactions with host components.
5. It is a widespread misconception that the described luciferase assay detects in vivo protein-protein interactions because the analysis involves tissue disruption and it remains unclear whether the protein interactions occur after cellular disintegration. This needs to be changed in l. 315 and throughout the manuscript. It's an in planta assay, but not an in vivo assay.

Version 1:

Reviewer comments:

Reviewer #1

(Remarks to the Author)

The revised manuscript made significant revision based on the comments of the reviewers. The authors performed additional analysis and experiments, and provided strong new information to support their findings. I am satisfied with the scientific meaning and content of the revision. I have no further comments for the revised manuscript.

Reviewer #2

(Remarks to the Author)
I am largely satisfied with the author's revision. They have conducted additional experiments that strengthen their claims (e.g. testing co-expression of WTK4 and AvrWTK496224 in one additional Ae. tauschii line; testing virulent Avr variant Bgt-55150AvrWTK4_3 and the non-recognized effector AvrPm3b2/c2). Additionally, they eliminated statements that AF 'confirmed' some of the modelled interaction interfaces. The new Supplementary Table SN1 provides more detail on the modelling and acknowledges the variability. It is now clearer that AF cannot distinguish between WTK4 interactions with virulent and avirulent variants of the corresponding effectors.

Two points remain to be fixed:

1. Line 419ff:“Strikingly, two independent AvrWTK4 mutants have the exact same mutation in Bgt-55150, resulting in the T30N substitution at the protein level, a mutation that is also present in the naturally virulent isolate THUN-12. Structural modelling and interaction assays showed that this mutation strongly reduces interaction with the HMA domain of WTK4 (Fig. 4; Supp. Note).“

Neither the structural modelling, nor the interaction assays were performed with a AvrWTK4 variant carrying only a T30N substitution (e.g. AvrWTK4-3), but with AvrWTK4THUN12 which contains two additional aa polymorphisms. Thus, there is no data to support that this mutation specifically affects the interaction with the HMA domain of WTK4. Furthermore, as the authors state, AF modelling cannot yet distinguish between potential differences in protein interactions caused by single amino acid substitutions. Therefore, structural modelling is an inappropriate method with which to assess the impact of T30N on interaction.

2. In Supp Table SN1, the forward slash „/“ is missing between 0.482 0.292.

Open Access This Peer Review File is licensed under a Creative Commons Attribution 4.0 International License, which permits use, sharing, adaptation, distribution and reproduction in any medium or format, as long as you give appropriate credit to the original author(s) and the source, provide a link to the Creative Commons license, and indicate if changes were made.
In cases where reviewers are anonymous, credit should be given to 'Anonymous Referee' and the source.
The images or other third party material in this Peer Review File are included in the article’s Creative Commons license, unless indicated otherwise in a credit line to the material. If material is not included in the article’s Creative Commons license and your intended use is not permitted by statutory regulation or exceeds the permitted use, you will need to obtain permission directly from the copyright holder.
To view a copy of this license, visit https://creativecommons.org/licenses/by/4.0/

Revisions to manuscript NCOMMS-25-71472A: An HMA-like integrated domain in the wheat tandem kinase WTK4 recognises an RNase-like pathogen effector.

Dear editors, dear reviewers,

Thank you very much for the evaluation of our manuscript submitted to *Nature Communications*. We are grateful for your constructive comments and suggestions, which have helped us improve the manuscript.

We have carefully considered all reviewers' recommendations. We have addressed all comments related to lack of clarity, imprecise statements, and requests for further clarification. We have removed Supp. Fig. S6 and replaced it with a Supplementary Note containing more relevant and important details regarding the AlphaFold modelling (located in a separate file). We also updated Fig. 3, Fig. 4, Supp. Fig. S3 and Supp. Fig. S7 (newly named S6) with corrections and results from new experiments and analyses. After performing the additional requested experiments, we made minor modifications to the Author contribution section. We also updated the Acknowledgement section and the affiliations. The names and order of the authors are the same as in the original manuscript.

We would like to note that completing the experimental work for this revision has been challenging due to the closure of Dr. Beat Keller's laboratory following his retirement last year. This situation resulted in significant constraints, including highly reduced manpower and financial resources. Despite these limitations, we have made every effort to address the comments requesting additional experiments. All changes are detailed in the point-by-point response below.

Finally, for consistency with nomenclature used in our recent publications (i.e. Bernasconi, Herger, Caro, Kunz et al. 2026; https://doi.org/10.1038/s41477-025-02180-w), we have changed the *Avr* gene nomenclature throughout the text and the figures, by specifying the isolate name in superscript instead of subscript (i.e. $AvrWTK4^{96224}$ instead of $AvrWTK4_{96224}$).

All modifications have been made in a Microsoft Word document using track changes and are highlighted accordingly.

Thank you for considering this revised version for publication in *Nature Communications*.

Best regards,

Zoe Bernasconi and Beat Keller

Reviewer #1 (Remarks to the Author):

This manuscript identifies and characterizes AvrWTK4, the effector from wheat powdery mildew recognized by the tandem kinase protein (TKP) immune receptor WTK4. The authors use a combination of genetic mapping, mutagenesis, biochemical interaction assays, and structural modeling approaches. The discovery of AvrWTK4 and the mechanistic characterization of its interaction with the HMA-like domain of WTK4 is a substantial contribution to the field of plant immunity. The work is timely, novel, and provides a clear mechanistic model for how TKPs can recognize effectors via integrated decoy domains, paralleling the established mechanism in NLRs. The experiments are well-designed, the data are robust, and the conclusions are supported by the evidence.

Comments & Suggestions:

1. The current title can be revised more directly to state the core mechanistic finding. For example: "A wheat tandem kinase immune receptor recognises a powdery mildew effector via an integrated HMA-like decoy domain", or "Direct effector recognition by a tandem kinase protein is mediated by an integrated HMA-like domain"

We agree that the title could be revised to more directly reflect the core mechanistic finding. However, we also consider reviewer #2's comment stating that the evidence for a direct effector-receptor interaction is currently limited. While we appreciate the suggested titles, we feel they may overstate the strength of the data. In addition, the first proposed title is longer than the *Nature Communications* guidelines allow. For these reasons, we suggest keeping the original title, while remaining open to further modifications that take both reviewers' comments into account.

2. Adjust the abstract's final sentence from "These findings further highlight..." to a stronger statement like: "We establish that an integrated HMA-like domain serves as the direct effector-binding decoy for WTK4, providing a mechanistic paradigm for effector recognition by tandem kinase immune receptors."

We revised the abstract to include a more mechanistic final sentence inspired by your suggestion:

- Lines 47-48: "We found that the integrated HMA-like domain possibly serves as a direct effector-binding decoy for WTK4, providing a mechanistic paradigm for effector recognition by tandem kinase proteins. ~~These findings further highlight that integrated domains in tandem kinase proteins serve as decoys for pathogen effectors, which could be leveraged to design novel recognition specificities.~~"

However, considering reviewer #2's concern about potentially overstating WTK4 as a direct sensor, we remain open to modifying the sentence to balance mechanistic clarity with appropriate caution, thereby addressing the concerns of both reviewers.

3. Line 79: When mentioning Bgt Avrs (e.g., AvrPm2, AvrPm3), add a brief note that these are NLR-recognized to contrast with the TKP-recognized AvrWTK4 (the manuscript's focus).

We modified the sentence to put the emphasis on the fact that these Avr are recognised by NLRs:

- Line 83: "Notable examples of NLR-recognised Avr effectors include the AvrPm2 and AvrPm3 effectors in *Bgt* (Bourras et al. 2015; Bourras, Kunz, Xue, Praz, Muller, et al. 2019; Praz et al. 2017), and the Avra effectors in *Bh* (X. Lu et al. 2016), all exhibiting a typical RNase-like structure."

4. Line 167-168: The exception of AvrWTK4_5 being virulent on Pm3f is noted but not discussed. A brief sentence explaining that this is likely due to an unrelated, secondary mutation would be helpful.

We added a brief explanation of the AvrWTK4_5 gain of virulence, the cause of which could not be identified:

- Lines 175-179: "The cause of this gain of virulence is currently unclear, since mutant AvrWTK4_5 does not have mutations in AvrPm3$^{a2/f2}$ or SvrPm3, nor genes described as effector regulators, which could be linked to a *Pm3f*-virulence phenotype (**Supp. Tables S4-S5**). Therefore, it is probable that an unknown mutation is responsible for the observed phenotype"

5. Line 206-208: Address "unknown mutation" in AvrWTK4_5: The manuscript notes AvrWTK4_5 has reduced AvrWTK4 expression but no mutations in Bgt-646/AvrWTK4. Add a brief statement about exploratory analyses (e.g., "We screened for mutations in 10 known effector regulators (e.g., XYZ; Bernasconi et al. 2024) but found none; future RNA-seq or ChIP-seq will test for indirect regulatory cascades"). This avoids leaving the gap unaddressed.

In the Results and Discussion sections, we state that we examined the mutations present in the AvrWTK4_5 isolate; however, none could be linked to AvrWTK4 or Bgt-646 (i.e. new lines 218-221). To our knowledge, Bgt-646 is the only described regulator of *AvrWTK4* expression in *B. graminis* f. sp. *tritici*. However, we agree that this point requires clarification. We modified the corresponding paragraph in the Discussion to better clarify this aspect:

- Lines 445-454: "Intriguingly, in the case of the mutant AvrWTK4_5, the mutation causing the virulence phenotype remains unknown, as no mutations were found in either *AvrWTK4* or *Bgt-646*. Furthermore, comprehensive analysis of all sequence polymorphisms in the AvrWTK4_5 mutant failed to identify any mutation in known or predicted genes linked to expression regulation (**Supp. Tables S4-S5**). This is consistent with our previous study, in which we could not find the phenotype-causing mutation for three out of seven *Pm3a/WTK4* virulent mutants (Bernasconi et al. 2024). This suggests a more complex regulatory network controlling *AvrWTK4* expression and points to additional, yet unidentified, components involved in modulating avirulence on *WTK4*. Future transcriptomic or chromatin-based analyses (e.g., RNA-seq or ChIP-seq) will be needed to determine whether the gain of virulence in these mutants results from indirect regulatory effects or epigenetic changes."

6. The current data showing that cell death requires an Ae. tauschii-specific component (Fig. 3, Supp. Fig. S3) is intriguing but currently feels like an unresolved thread. Co-expression of Ae. tauschii-derived helper NLR candidates (e.g., orthologs of WTK3 and Sr62's helper NLR WTN1/Sr62-NLR; Lu et al. 2025; Chen et al. 2025) may be texted in N. benthamiana. Even negative data will strengthen the conclusion that WTK4 requires a co-factor that is not a common canonical helper NLR."

The discussion (lines 415-424) correctly links this to the emerging theme of helper NLRs for TKPs. In the Results section (line 261-265), after presenting the negative data in N. benthamiana and wheat, try to add a sentence like: "This suggests that WTK4-mediated cell death requires an additional host-specific factor, consistent with recent models where TKPs act as sensors that rely on helper proteins for full immune

activation (Chen et al., 2025; Lu et al., 2025)." This frames the finding as part of an active area of research rather than a limitation.

We tested whether WTK4-mediated cell death could be restored by co-expression with a helper NLR by performing co-expression assays of WTK4 and AvrWTK4 together with $SR62^{NLR}$, the helper NLR associated with the TKPs SR62/RWT4 (new **Supp. Fig. S3**). Due to technical constraints, we could only use the helper NLR allele derived from the wheat cultivar Fielder, as the *Aegilops sharonensis* NLR is toxic in *E. coli*, making cloning challenging, as previously reported (Chen et al. 2025). Importantly, the Fielder-derived $SR62^{NLR}$ functions efficiently as a helper for both SR62 and RWT4 (Chen et al. 2025, Lu et al. 2025).

Consistent with our protoplast assays in Fielder, which did not show HR upon co-expression of *WTK4* and *AvrWTK4* (new **Supp. Fig. S3C**), we did not observe HR in *N. benthamiana* in the triple co-expression assay (new **Supp. Fig. S3A**). By contrast, the positive control combination AvrSR62-$SR62^{TK}$-$SR62^{NLR}$ induced a robust HR. These results indicate that WTK4-mediated cell death requires an additional host-specific factor that is not provided by this canonical helper NLR.

We have added several sentences in the Results and in the Discussion sections, and modified **Supp. Fig. S3** to include the new data:

- Lines 279-284: "Because other TKPs, such as $SR62^{TK}$ and RWT4, share the same helper NLR required for inducing HR in heterologous systems, we wondered whether *WTK4-Bgt-55150*^{96224} cell death could also be triggered by co-expression of the same helper NLR ($SR62^{NLR}$). We performed co-expression assays using the Fielder-derived *SR62*^{NLR} version, which successfully induced HR when co-expressed with *AvrSR62* and *SR62*^{TK}. In contrast, co-expression of *WTK4-Bgt-55150*^{96224}*-SR62*^{NLR} did not induce HR in *N. benthamiana*, indicating that $SR62^{NLR}$ is not a helper of WTK4 (**Supp. Fig. 3A-B**)".
- Lines 462-465: "Notably, co-expression of WTK4-AvrWTK4 with $SR62^{NLR}$, the helper NLR required for the TKPs $SR62^{TK}$ and RWT4, failed to induce cell death in *N. benthamiana*. This indicates that WTK4 function does not depend on this known helper and likely requires a distinct one.".
- Lines 24-30 of the Supp. Figures file: "**Supp. Fig. S3. Co-expression of AvrWTK4^{96224} ~~(Bgt-55150_96224)~~ and WTK4 does not lead to cell death in ~~(A)~~ N. benthamiana or ~~(B)~~ wheat protoplasts (wheat cv. Fielder).** (A) CLUC_WTK4 was co-infiltrated in *N. benthamiana* either alone, with NLUC_AvrWTK4^{96224} or NLUC_avrWTK4$^{THUN\text{-}12}$ (infiltrations 1-3). In infiltrations 4 and 5, $SR62^{NLR}$ was added to CLUC_WTK4 and either NLUC_AvrWTK4^{96224} or NLUC_avrWTK4$^{THUN\text{-}12}$. As positive control, $SR62^{NLR}$ was co-infiltrated with $SR62^{TK}$ and AvrSR62-7 (infiltration 6). No cell death was observed for any infiltration except for the positive control. The cell death signal was measured 5 days post-infiltration."

We also added an explanatory sentence at the end of the Results section, inspired by the one suggested by the reviewer:

- Lines 293-294: "These findings indicate that at least one genetic component unique to *Ae. tauschii* and absent in *N. benthamiana* or the wheat cultivar Fielder is needed for inducing HR upon recognition of *Bgt-55150*^{96224}, consistent with recent models where TKPs act as sensors that rely on helper proteins for full immune activation (Chen et al., 2025; Lu et al., 2025)."

7. In the text (lines 306-308), explicitly state what the "linker sequence" is (e.g., "the ~XX amino acid segment connecting the HMA domain to the K1 pseudokinase domain").

We added a description of the linker sequence in the text:

- Lines 344-347: "The model depicted two main interaction surfaces on WTK4: one involving the β2 sheet of HMA_{WTK4}, and one the linker sequence between HMA_{WTK4} and the pseudokinase domain (amino acids 69-104; **Fig. 4B**)."

8. Line 372: "It is noteworthy that" –> This is a slightly weak transition. Consider "Strikingly," or "A key finding is that".

We changed the text as suggested.

- Line 419: "~~It is noteworthy that~~ Strikingly, two independent *AvrWTK4* mutants have the exact same mutation in *Bgt-55150*, resulting in the T30N substitution at the protein level..."

9. Line 425: "represents a significant advance" –> This is an opinion. It's better to let the data speak for itself. Consider rephrasing to: "The discovery of an HMA-like ID in WTK4 provides a mechanistic basis for..."

We changed the text as suggested.

- Lines 489-491: "The discovery of an HMA-like ID in WTK4 ~~represents a significant advance in understanding the mechanistic basis of~~ provides a mechanistic basis for studying resistance conferred by TKPs, a novel class of immune receptors."

10. Line 439: "MAX-like effectors in its repertoire". Specify "B. graminis f. sp. tritici" instead of "its."

In the text, the sentences reads: "*WTK4* confers resistance against wheat powdery mildew, a pathogen which lacks MAX-like effectors in its repertoire (Seong and Krasileva 2023, 2021). Its corresponding effector, AvrWTK4, belongs to the RNase-like family", therefore we feel that replacing "its" in the first sentence with "*B. graminis* f. sp. *tritici*" would be a repetition, due to the presence of "wheat powdery mildew" earlier on. In the second sentence, "Its" is more referred to WTK4. However, we would replace "wheat powdery mildew" with "*B. graminis* f. sp. *tritici*", to be consistent with the Latin naming ("M*agnaporthe oryzae*") used in the previous sentence:

- Lines 503-504: "*WTK4* confers resistance against *B. graminis* f. sp. *tritici*, a pathogen which lacks MAX-like effectors in its repertoire".

11. In the discussion (around lines 443-447), briefly speculate on how the HMA domain of WTK4 evolved to recognize this specific RNase-like effector, unlike the HMA domains in rice that recognize MAX effectors. Is the binding surface fundamentally different? This reinforces the potential for engineering and sets the stage for future work.

We thank the reviewer for this insightful suggestion. To address this point, we compared the interaction surfaces between different HMA domains and their corresponding Avr effectors. Based on this, we added few sentences to the Discussion that highlight potential differences in binding interfaces and emphasise the implications for engineering novel recognition specificities. To improve readability and narrative flow, this new information was incorporated into the existing paragraph discussing the diversity of effectors recognized by HMA domains and the potential applications of IDs, with slight rephrasing of the surrounding text. We also added a short sentence in the Results part highlighting that the AvrWTK4-WTK4 interaction is predicted to happen through the β2 sheet of HMA_{WTK4}:

- Lines 345-347: "The model depicted two main interaction surfaces on WTK4: one involving the β2 sheet of HMA_{WTK4}, and one the linker sequence between HMA_{WTK4} and the pseudokinase domain (amino acids 69-104; **Fig. 4B**)."
- Lines 516-538: "~~It remains to be explored~~ For future applications, it will be important to determine whether different effector types bind overlapping or distinct surfaces on HMA domains. AVR1-CO39 binds HMA_{RGA5} primarily through its α1/β2 surface. In contrast, AVR-PikD binds HMA_{Pikp-1} via its β2/β3/β4 surfaces. These distinct binding interfaces have been successfully combined in engineered HMA domains to confer dual recognition specificity (Cesari et al., 2013; Maqbool et al., 2015; Cesari et al., 2022). The *M. oryzae* effector Pwl2 binds $HMA_{OsHIPP43}$ through an extensive interface involving mainly α1, β3, and the loop between α1 and β2, and this knowledge has also been used to design a synthetic receptor with broad-spectrum resistance (Zdrzalek et al., 2024). A recent study presented the targeted engineering of the HMA-like domain of the tandem kinase protein Rmo2 to recognise the wheat blast effector PWT7, in addition to the naturally recognised PBY2, highlighting the potential of this approach in TKPs (Yu et al. 2025). In this study we predicted, and then experimentally showed, that AvrWTK4 binds to HMA_{WTK4} primarily through its β2 surface, and that modifications of three residues in the β2 surface is enough to disrupt AvrWTK4 binding (**Fig. 4**). Such knowledge could potentially be used to design synthetic HMA modules capable of recognising a broader range of Avr effectors, even from unrelated pathogens, such as *Bgt* and *M. oryzae*. ~~Here, we predicted and then experimentally confirmed a specific interaction between HMA_{WTK4} and the avirulent AvrWTK4 variant, but not with the virulent variant, illustrating the potential of AlphaFold for *in silico* analysis of recognition specificity (**Supp. Fig. S6**). So far, engineering efforts have shown that altering a few residues in the HMA domain of RGA5 to mimic those of Pikp-1 enables RGA5 to recognise AVR-Pik, thereby broadening its recognition specificity (Cesari et al. 2022). Similarly, replacing the HMA domain of Pikp-1 with that of a natural host target, HIPP43, conferred broad-spectrum resistance (Zdrzalek et al. 2024).~~ In parallel, nanobody-based IDs have been introduced into NLRs to expand recognition potential, offering another promising approach for engineering immune receptors (Kourelis et al. 2023)."

12. Clarify phenotyping details: In bi-parental mapping, specify how the 0–100 virulence score was calibrated (e.g., "% leaf area covered by mycelium, with three technical replicates per leaf segment") and whether inter-observer consistency was assessed. This strengthens the QTL mapping validity.

We did not formally assess inter-observer consistency because the differences in virulence phenotypes were generally clear. However, to ensure reproducibility and consistency, for phenotyping we used a minimum of three leaf segments, each coming from a different plant (biological replicates) for each isolate. We have now added a sentence in the Methods section clarifying this procedure (for consistency, we also added the same information to the next section, "Mutagenesis and phenotyping experiments").

- Lines 574-575: "To ensure consistency, all leaf area coverage scores were evaluated by a single trained investigator based on at least three independent biological replicates."
- Lines 596-597: "Disease severity was assessed by eye 7-8 days after inoculation for at least three biological replicates per line, and the virulence was based on the percentage of the leaf area covered by the fungal mycelium."

13. Validate structural model confidence: The AlphaFold Multimer results (ipTM 0.73–0.86) are strong, but add pLDDT scores for the interaction interface residues (e.g., "Residues I29/D30/Q31 in HMAWTK4 and T30 in AvrWTK496224 had pLDDT > 90, indicating high structural confidence"). This supports the functional relevance of the predicted interface.

Following this as well as reviewer #2's comments on the AlphaFold modelling, we have added a new Supplementary Note that provides additional details on the AlphaFold predictions of the AvrWTK4-WTK4 interaction. In this section, we have also included a table reporting the pLDDT values for the key interacting residues, as suggested (see **Supp. Table SN3**). These values are consistently high (pLDDT > 80 for most predictions), indicating high structural confidence for the residues of the predicted interaction interface and supporting the functional relevance of the model. We have added a sentence in the Results section highlighting this finding, and a reference to the new section in the Methods section:

- Lines: 347-349: "The main residues predicted to be involved in the interaction surfaces have pLDDT values consistently above 68 for AvrWTK4 and 82 for WTK4, reflecting high confidence in the modelled interface (**Supp. Note**)."
- Lines 744-745: "Details on the AvrWTK4-WTK4 interaction modelling can be found in **Supp. Note**."

14. Expand on the finding that AvrWTK4 (RNase-like) binds HMAWTK4, whereas NLR HMA domains bind MAX effectors: "This convergence—distinct effector families targeting HMA domains—suggests HMA-like IDs are 'hotspots' for pathogen manipulation. Unlike NLR HMA domains, which co-evolve with MAX effectors (Seong & Krasileva 2021), HMAWTK4's interaction with RNase-like AvrWTK4 may reflect independent adaptation to powdery mildew."

We added the sentence as suggested, with some minor modifications, and adapted the rest of the text to avoid redundancy.

- Lines 508-515: "This shows that effectors from different pathogen species have convergently evolved to target and bind HMA domains from their hosts. This convergence, where distinct effector families target HMA-like domains, suggests that these domains are "hotspots" for pathogen manipulation. Furthermore, HMA-like IDs appear capable of evolving diverse effector-binding specificities, possibly through minimal sequence changes. Unlike NLR HMA-like IDs, which co-evolve with MAX effectors (Seong & Krasileva 2021), the interaction between HMA_{WTK4} and the RNase-like AvrWTK4 effector likely reflects an independent adaptation to the powdery mildew pathogen. ~~This, in turn, suggests that HMA(-like) domains integrated in immune receptors might be capable of evolving diverse effector-binding specificities, possibly through minimal sequence changes~~."

15. Supp. Fig. S1: Add Virulent (+)/Avirulent (-) entries for each isolate-R gene combination in the Table.

We added the suggested information in **Supp. Fig. S1** and modified the figure legend accordingly.

- Lines 11-12 of the Supp. Figures file: "The symbol on the left side of every column indicates a virulent (+) or an avirulent (-) phenotype.".

Reviewer #2 (Remarks to the Author):

The manuscript by Zoe Bernasconi et al. reports the isolation of the avirulence effector AvrWTK4 from the wheat powdery mildew pathogenic fungus, which is recognized by the wheat tandem kinase (TKP) ‚immune sensor' WTK4. The authors identified AvrWTK4 through the use of two independent methods, a bi-parental powdery mildew mapping population and a UV mutagenesis-based approach and show that the gene encodes a deduced RNase-like protein (RALPH). They also present evidence that another powdery mildew gene, Bgt-646, encoding a predicted ankyrin-repeat containing protein, is required for AvrWTK4 expression and thus mutants in Bgt-646 render an AvrWTK4 containing isolate virulent on WTK4 containing wheat. They then present evidence that co-expression of AvrWTK4 with WTK4 induces a mild cell death phenotype only in Ae. tauschii protoplasts. AlphaFold-based structural models suggests that AvrWTK496224 interacts with a previously unnoticed HMA domain at the N-terminus of WTK4 while the virulent variant of AvrWTK496224 (avrWTK4THUN-12) does not. Limited experimental support for this is provided by split-luciferase experiments in planta and yeast two-hybrid experiments. These experiments show that AvrWTK496224 interacts with the HMA+linker region, while a much weaker interaction with full-length WTK4 is observed.

Overall, the study presents an important advance in understanding the function of a TKP in grasses in an isolate-specific plant immune response. Text and Figures are well organized and many of the claims of the manuscript are supported by experimental evidence.

I have the following major comments

1. A key observation by the authors is that co-expression of AvrWTK4 with WTK4 induces a mild cell death in Ae. tauschii protoplasts (33% reduction in relative fluorescence intensity; Fig. 3D and 3E), but not in N. benthamiana and hexaploid wheat (cv. Fielder). Given the mild cell death phenotype detected, it will be important to substantiate the specificity of the effector-triggered and WTK4-mediated (and indirectly inferred) cell death using the virulent version AvrWTK4-3 (or AvrWTK4-6 leading to a T30N substitution in the deduced protein) described in this study. It will also be important to extend these experiments to a known Bgt RALPH effector belonging to the same RNase-like subfamily as AvrWTK4 and showing the greatest sequence similarity to this avirulence effector.

We have repeated the protoplast assays as suggested, including the virulent Avr variant Bgt-55150^{AvrWTK4_3} (identical to the Bgt-55150^{AvrWTK4_6} variant) and the non-recognised effector AvrPm3$^{b2/c2}$. AvrPm3$^{b2/c2}$ belongs to the same effector family as Bgt-55150 (E008), and has the highest sequence similarity to it, together with Bgt-51460, which is, however, not expressed in CHE_96224, and was therefore considered less appropriate (see new **Fig. 3**, and new **Supp. Fig. S3**).

Additionally, we replaced the empty vector control with a GUS-expressing construct as negative control, to ensure that all treatments involved the expression of a non-native protein, providing a more accurate baseline for cell viability.

For consistency, we also tested the suggested controls in a protoplast cell death assay in wheat cv. Fielder, which is included and substituted the old data in **Supp. Fig. S3**.

In these expanded experiments, neither the virulent variant Bgt-55150^{AvrWTK4_3} (carrying the T30N mutation) nor the AvrPm3b negative control induced a significant reduction in viability ($p > 0.5$) compared to the GUS or WTK4-only controls in the *Ae. tauschii* lines TOWWC0111 and TOWWC0167 (new **Fig. 3**). A marginal shift was observed in TOWWC0167 when co-transfecting *WTK4* and *AvrPm3*$^{b2/c2}$. We attribute this marginal reduction in cell viability to the inherent variability of this system. Importantly, the *AvrPm3*$^{b2/c2}$ control was statistically indistinguishable from the *GUS/WTK4* control, and the non-recognised *Avrs* ($p > 0.05$), supporting the consistency of our negative controls (for statistical details see **Supp. Table S6**).

The *Bgt-55150*$^{AvrWTK4\_3}$ allelic variant showed intermediate YFP fluorescence intensity, between *Bgt-55150*^{96224} and *Bgt-55150*$^{THUN-12}$ in TOWWC0111 (new **Fig. 3D**). However, it was not significantly different from *Bgt-55150*$^{THUN-12}$, nor from *GUS* and *AvrPm3*$^{b2/c2}$, in any of the tested lines and combinations, further indicating that the T30N mutation present in both Bgt-55150^{AvrWTK4_3} and Bgt-55150$^{THUN-12}$ is likely the main determinant of virulence (see new **Fig. 3D-E**).

In the repeated protoplast experiments, *AvrWTK4*^{96224} transfection, as previously observed, induced only a mild cell death in presence of *WTK4*. We note that the effect of *AvrWTK4*^{96224} transfection was slightly less pronounced than in the original experiment, possibly due to biological variation inherent to the protoplast system (e.g., experiments performed in winter, by different researchers, and with new biological material and chemicals). To ensure a robust analysis, we applied a standardised pipeline, including within-experiment normalisation and outlier removal (values outside the median ± 2*IQR). An approach of multiple comparisons of means (contrast analysis) following one-way ANOVA, which had increased statistical power compared to our previous statistical approach, was used. This is now described in the Methods section. Following these processing steps, a statistically significant reduction in cell viability (~15-20%) was consistently observed upon *AvrWTK4*^{96224} expression compared to *GUS*, *Bgt-55150*$^{AvrWTK4\_3}$, and *Bgt-55150*$^{THUN-12}$. This effect was consistently observed in two settings: in TOWWC0111, which has native *WTK4* expression, and in TOWWC0167 only when co-transfected with *WTK4* (blue bars in new **Fig. 3D-E**).

These results collectively substantiate that the observed cell death is specific to the recognition of AvrWTK4^{96224} by WTK4.

Fig. 3 and its legend have been modified to include the new experiments. The main text has been modified to include the new data (see also our response to the next comment for a full description of the textual changes):

- Lines 261-264: "The *Bgt-55150* gene sequences of CHE_96224 (avirulent), mutant AvrWTK4_3 and THUN-12 (both virulent) were codon-optimised for plant expression and cloned into expression vectors without signal peptide. We selected *AvrPm3*$^{b2/c2}$ as negative control. It encodes an RNase-like effector which is most closely related to *Bgt-55150* at sequence level."
- Lines 699-706: "Within each experiment, the obtained values were normalised (i) to the median value of the GUS (TOWWC0111) or WTK4+GUS (TOWWC0167) treatment, and (ii) to the median value of a treatment set (i.e. values of biological replicate "a" for all treatments represents the treatment set "a"). Measurements outside of the median ± 2 * interquartile range (IQR) were considered as outliers and removed from the analysis. The experiment was repeated three times for TOWWC0111 (~~for~~ nine

biological replicates in total), and four times for TOWWC0167 (twelve biological replicates in total). A one-way ANOVA followed by multiple comparisons of means (contrast analysis) was used to estimate significant differences between treatments."

2. In the discussion the authors conclude that 'together, these findings support a broader model in which TKPs act as sensors that depend on helper proteins (possibly NLRs) for full immune activation' (ln 420-422). An alternative model is that WTK4 is not an immune sensor, but instead a putative Ae. tauschii lineage-specific sensor NLR indirectly detects manipulation of WTK4 by AvrWTK4. I mention such an alternative model also because the data claiming a direct interaction between AvrWTK4 and full-length WTK4 are not conclusively convincing (e.g. undetectable in yeast two-hybrid experiments; Fig. 4E). If the helper NLR model proposed by the authors is correct, it would be expected that protoplasts of phylogenetically divergent Ae. tauschii genotypes would all be capable of mediating cell death upon co-expression of WTK4 and AvrWTK4, since helper NLRs are typically conserved in a phylogenetic plant lineage. This will also clarify whether the incidence of effector-triggered and WTK4-mediated cell death is quantitatively similar or dissimilar in phylogenetically distant Ae. tauschii lines. These additional experiments can be conducted in a reasonable time frame and the discussion need to be modified accordingly. Based on the data presented, the authors cannot claim that WTK4 is a 'wheat tandem kinase immune receptor' (lines 39–40 in the abstract and throughout the manuscript).

We thank the reviewer for this insightful comment regarding the distinction between the proposed "helper NLR" model and the alternative "sensor NLR monitoring WTK4" model. The reviewer's point refers to a statement in the Discussion section, where we aimed to place our findings in the broader context of TKPs. We would like to clarify that our study does not directly address the identity of the putative additional component required for immune activation, but instead focuses on the identification and characterisation of AvrWTK4.

In response to this comment, we performed additional experiments using the susceptible *Aegilops tauschii* lineage 2 (L2) accession TOWWC0167, which lacks a functional *WTK4* allele. As shown in the newly added **Fig. 3E**, co-expression of *WTK4* and *AvrWTK4*^{96224} induces cell death in this L2 background. This response is comparable to that observed in a resistant, *WTK4*-containing L2 accession (new **Fig. 3D**), but contrasts with our results in hexaploid wheat (cv. Fielder), where no cell death was detected (**Supp. Fig. S3**). These findings indicate that the required additional component is present in multiple L2 backgrounds, irrespective of their endogenous *WTK4* status.

We agree with the reviewer that extending this analysis to phylogenetically divergent *Ae. tauschii* lineages would provide valuable insight into the conservation of this component. However, such experiments are currently limited by the availability of non-L2 germplasm, and the time required to generate sufficient material. We also consider this beyond the scope of the present study. Nevertheless, we have revised the Discussion to clarify that it remains unresolved whether the missing component is species-wide or L2-specific.

Regarding the mechanistic model, we acknowledge that the alternative model proposed by the reviewer, where a lineage-specific sensor NLR monitors perturbations of WTK4 by AvrWTK4, remains plausible. Importantly, both this model and our originally proposed helper NLR model are consistent with the available data. We have therefore revised the Discussion to explicitly include this alternative hypothesis and to temper our interpretation accordingly.

Finally, we agree that referring to WTK4 as a standalone "immune receptor" may be an overstatement. Given the likely involvement of additional components, we have revised the manuscript to use more cautious

terminology and to better reflect that WTK4 is likely part of a receptor complex, consistent with current understanding of TKP-mediated immunity.

After inclusion of the additional experiments, and in light of the reviewer's comments, we made the following modifications in the manuscript:

- Lines 40-42: "In this work, we identify AvrWTK4, the wheat powdery mildew RNase-like effector recognised by the wheat tandem kinase ~~immune receptor~~ protein WTK4..."
- Lines 44-45: "Transfection of *AvrWTK4* specifically induced cell death in *WTK4*-~~containing~~expressing *Aegilops tauschii* protoplasts."
- Lines 266-275: Co-transfection of *Bgt-55150*^{96224} with a YFP marker in the *Ae. tauschii* line TOWWC0111 significantly reduced YFP fluorescence intensity ~~by 33%~~ compared to *GUS*, *Bgt-55150*$^{THUN\text{-}12}$ and *AvrPm3b* co-transfected with the YFP marker (**Fig. 3D**). In TOWWC0167, no significant difference in relative YFP fluorescence intensity was observed between protoplasts transfected with either ~~an empty vector~~ GUS, *Bgt-55150*^{96224} or any other effector gene tested (**Fig. 3E**). However, when *WTK4* was additionally transfected in TOWWC0167, a significantly reduced YFP fluorescence intensity, indicative of cell death, was observed in combination with *Bgt-55150*^{96224} but not with the other tested effectors (**Fig. 3E**). These results indicate that *Bgt-55150*^{96224} induces cell death specifically in the presence of *WTK4* in *Ae. tauschii*. ~~From these results we~~ We therefore conclude that *Bgt-55150*^{96224} is *AvrWTK4."*
- Lines 303-313 (legend of **Fig. 3**): "(**D**) Protoplasts derived from TOWWC0111 and (**E**) TOWWC0167 were co-transfected with 3 pmol of a vector encoding YFP and 3 pmol of either ~~an empty vector~~ GUS, *Bgt-55150*^{96224} (avirulent allele), *Bgt-55150*$^{AvrWTK4\text{-}3}$, *Bgt-55150*$^{THUN\text{-}12}$ (virulent alleles), or *AvrPm3*$^{b2/c2}$ (negative control). For TOWWC0167, either *GUS* (grey bars) or *WTK4* (blue bars) was additionally transfected. The *Avr-R* pair *AvrSr35+Sr35* was used as a positive control. YFP fluorescence was measured ~~18~~16-20 hpi for two technical and three biological replicates per treatment. The experiment was repeated three times ~~for a total of 9 biological replicates~~ for TOWWC0111 and four times for TOWWC0167. Fluorescence values were normalised to the average fluorescence of protoplasts co-transfected with the *YFP* vector and ~~the empty vector~~ either *GUS* (TOWWC0111) or *WTK4* (TOWWC0167) for each experiment. Significance and p values (significance threshold of p < 0.05) were calculated with a ~~two~~one-sided analysis of variance (ANOVA) followed by ~~a Tukey HSD test~~ multiple comparisons of means (contrast analyses) with Bonferroni correction (***P ≤ 0.001; **P ≤ 0.01; *P ≤ 0.05; •P ≤ 0.10; n.s., not significant; **Supp. Table S6**)."
- Lines 383-386: "...virulent *Bgt* isolates evade recognition either through transcriptional downregulation of *AvrWTK4*, or via point mutations that specifically affect its HMA-binding interface, reducing direct interaction with the immune ~~receptor~~ sensor."
- Lines 475-486: "The fact that HR could be observed in an *Ae. tauschii* line from lineage 2 upon co-transfection of *WTK4* and *AvrWTK4* (**Fig. 3**), but not in wheat cultivar Fielder (**Supp. Fig. S3**), indicates that the putative helper of WTK4 is private to *Ae. tauschii*. TKPs and their NLR partners are frequently genetically linked and co-evolve, so they can be lost individually or together, with a consequent loss of resistance, even within the same species or lineage (Yue, Liu, and Liu 2025). However, to determine whether the putative helper of WTK4 is lineage- or species-specific, additional experiments involving *Ae. tauschii* lines outside lineage 2 would be required. ~~These findings~~ Our data, together with the existing literature, support a broader model in which TKPs act as sensors that depend on helper proteins (possibly NLRs) for full immune activation. Alternatively, it is also possible that AvrWTK4 manipulates WTK4 in a way that is sensed by a putative "guard" protein, which is required to trigger downstream signalling and the immune response."

3. In Line 264-266, the authors state that "These findings indicate that at least one genetic component unique to Ae. tauschii and absent in N. benthamiana or the wheat cultivar Fielder is needed for inducing HR upon recognition of Bgt-5515096224." To substantiate this statement, protein detection should be performed for the N. benthamiana cell death assay, as the absence of cell death upon co-expression of WTK4 and AvrWTK4 may also be explained by low accumulation of one of the two proteins. Additionally, whereas the protoplast assays performed in cv. Fielder contain the appropriate controls, the protoplast assays shown in Figure 3D should also contain at least one positive control (e.g the Sr50-AvrSr50 pair).

We thank the reviewer for this suggestion. We performed protein detection assays in *N. benthamiana* and confirmed that both the effectors and *WTK4* are expressed in this system, although WTK4 accumulated at relatively low levels. We optimized WTK4 detection by testing various tags (FLAG, GFP, and CLUC) and observed that CLUC-tagged WTK4 provided the most robust signal in Western blots. In co-infiltrated samples (CLUC_WTK4 + NLUC_AvrWTK4), all proteins were detectable, albeit WTK4 at lower levels than the Avrs, and no cell death was observed (see new **Supp. Fig. S3**).

Furthermore, following the suggestion of reviewer #1, we also co-expressed $SR62^{NLR}$, the helper NLR of the $SR62^{TK}$/AvrSR62 pair, with WTK4 and AvrWTK4. Cell death was not observed in this combination either. Notably, the addition of $SR62^{NLR}$ did not alter protein accumulation of either WTK4 or AvrWTK4 compared to infiltrations without the helper $SR62^{NLR}$ (**Supp. Fig. S3**).

The title of the Methods section describing the protein detection procedure has been changed. The figure has been updated and the legend modified as follows:

- Line 727: "Protein detection for split-luciferase and cell death assays"
- Lines 732-733: "…total protein extract were separated in SDS polyacrylamide (PA) gels (7.5%, 10% or 12%)…"
- Lines 30-34 of the Supp. Figures file: "(B) Immunoblot analysis of WTK4 and AvrWTK4 constructs used in the cell death assay shown in panel A. Protein samples were prepared as described in the Methods section and separated on a 10% SDS-polyacrylamide gel. On the left side of the membrane, proteins from single infiltrations (2:1 construct:P19 ratio) are shown. On the right side, proteins from co-infiltrations (1:1:1 AVR:R:P19 ratio) are shown. The membrane was incubated with an anti-LUC antibody and imaged for 10 seconds"

Although we believe that, if the helper component is absent from wheat, as discussed previously (**Supp. Fig. S3**), then it is also likely to be absent from *N. benthamiana*, we agree that the relatively low accumulation of WTK4 in *N. benthamiana* could potentially limit its ability to trigger cell death. We acknowledge this limitation of the experimental system and have added this point in the revised manuscript.

- Lines 281-284: "In both *N. benthamiana* experiments, WTK4 accumulated at lower levels than the AvrWTK4 variants. It is possible that low protein levels could potentially limit its capacity to trigger strong HR. To complement the *N. benthamiana* experiments, we also performed a protoplast assay in wheat. ~~Additionally, p~~Protoplasts of the wheat cultivar Fielder..."

Finally, as suggested, we have included a positive cell-death control (*AvrSr35+Sr35*) in **Fig. 3D-E**. Because we did not have access to the previously used control *(Sr50+AvrSr50*) anymore, we employed this alternative,

which induces an even stronger cell death response. For consistency, this control was also used in the protoplast assay in wheat cv. Fielder (**Supp. Fig. S3C**).

- Lines 33-37 of the Supp. Figures file: "(C) The YFP fluorescence in wheat protoplasts was measured 18 ~~20~~ hours post-transfection and normalised to the fluorescence value of protoplast transfected with YFP ~~alone~~ with GUS. As a positive control, ~~Sr50~~ Sr35 co-transfected with its corresponding Avr effector ~~AvrSr50~~ AvrSr35 was used."

4. In line 318 the authors state that "AvrWTK496224 also interacted with full-length WTK4, but to a lesser extent, likely due to its lower protein levels (Fig. 4D ii, Supp. Fig. S7)." The samples need to run on the same gel/blot in order to compare protein levels between AvrWTK496224 and WTK4. In addition, the size marker needs to be shown, control lane needs to be included and full-length blots need to be provided. This experimental setup should be considered for Supp. Fig. S8 as well.

We apologise for the incomplete presentation of the Western blots. Actually, in **Supp. Fig. S7** (newly numbered as **S6**), AvrWTK4 and WTK4 were run on the same membrane. The membrane was cropped in two, because it was originally imaged at two different exposure times to allow for optimal visualisation of each protein (30 seconds for the AvrWTK4 constructs and two minutes for the WTK4 constructs). However, we agree that presenting the full images facilitates comparison of protein abundance. Therefore, we have now modified the presentation of the figure so that it shows both the WTK4 and AvrWTK4 constructs on the same membrane (new **Supp. Fig. S6**).

Samples for the HMA+linker construct were collected, prepared, and processed for immunoblotting at the same time and conditions as those used for AvrWTK4 and WTK4. However, due to the large difference in molecular weight between the proteins (WTK4: ~100kDa; HMA+linker: ~25kDa), the samples were separated on different gels: a 7.5% SDS-polyacrylamide gel for WTK4 and a 12% SDS-polyacrylamide gel for HMA+linker. The corresponding blot image has also been included in **Supp. Fig. S6**.

As shown in the new figure, the appropriate controls are present: a sample with P19-infiltrated leaf tissue and a molecular weight marker for each blot. The raw, uncropped membrane images will be deposited during the final steps of manuscript submission.

The figure legend has been updated as follows:

- Lines 76-86 of the Supp. Figures file: "LUC-tagged constructs used in the split-luciferase assay were infiltrated into *Nicotiana benthamiana* leaves along with P19, using a construct-to-P19 ratio of 2:1. Three days after infiltration, six leaf discs were collected from three different leaves, and total protein was extracted as outlined in the Methods section. The Avr, WTK4 full-length and K1+K2 constructs were loaded on a 7.5% SDS-Acrylamide gel, while the HMA+linker constructs on a 12% SDS-Acrylamide gel. The membrane containing the Avr, WTK4 full-length and K1+K2 constructs ~~were~~ was exposed for 2 minutes, while the membrane containing the HMA+linker constructs for 30 seconds. ~~The expected sizes in kDa are depicted in blue on the right of every blot. No significant expression difference was detected~~ Protein levels were comparable between WTK4 and $WTK4_{NoBind}$, HMA_{WTK4} and $HMA_{WTK4\text{-}NoBind}$, and $AvrWTK4^{96224}$ and $avrWTK4^{THUN\text{-}12}$. Below, the membranes were stained with a Ponceau solution as a loading control."

5. In line 353 the authors state "Residues involved in interactions are highlighted in grey and underscored." This needs to be corrected to "…predicted to be involved in interactions…". Only a high-resolution structure can provide conclusive evidence for this claim.

We rephrased the sentence as suggested (line 399).

6. In line 372 "Structural modelling and interaction assays confirmed that this mutation strongly reduces interaction with the HMA domain of WTK4". This statement must be toned down for the following reasons: when using AlphaFold3 (AF3) (Template setting cutoff date 3/2/2025), the interaction between the deduced WTK HMA domain and AvrWTK4(Thun12) produces a higher ipTM score compared to the interaction with AvrWTK496224. See the interactions predicted below:

HMA-linker + AvrWTK4Thun12 : ipTM = 0.82 pTM = 0.77

HMA-linker + AvrWTK496224 : ipTM = 0.81 pTM = 0.77

HMA + AvrWTK4Thun12 : ipTM = 0.9 pTM = 0.87

HMA + AvrWTK496224 : ipTM = 0.89 pTM = 0.87

HMARPG1(=1-73) + AvrWTK4Thun12 : ipTM = 0.89 pTM = 0.88

Thus, the structural modelling does not "confirm" this specific interaction. Similarly, in 326 the authors state "…confirming the functional relevance of this interface (Fig. 4D i-ii)." This needs to be changed to something along the lines "… providing evidence that suggests the predicted interface is functionally relevant…".

Moreover, in AF3, prediction of the complex between Full-length (FL) WTK4 and AvrWTK4 is consistently poor (ipTM 0.17; Template setting cutoff date 2/3/2025). Thus, it is necessary to describe the exact parameters used to obtain the predicted complex between FL WTK and AvrWTK4, as shown in main Figure 4. Are the predictions with FL WTK reproducibly higher than >0.6 ipTM with the parameters used by the authors?

We thank the reviewer for carefully examining the AlphaFold predictions. We repeated the modelling and observed discrepancies between predictions obtained with AlphaFold2 and with different versions of AlphaFold3. Using AlphaFold2 and AlphaFold3 in July 2025 (the version used to generate the original **Supp. Fig. S6**), the predicted interaction between avrWTK4$^{THUN-12}$ and WTK4 was substantially weaker than that between AvrWTK4^{96224} and WTK4 (ipTM 0.292 vs 0.73 in AlphaFold2, and 0.17 vs 0.62 in AlphaFold3, July 2025). However, this difference is no longer observed when using more recent AlphaFold3 versions.

Differences between AlphaFold3 predictions obtained at different time points likely reflect updates to the AlphaFold3 model and underlying sequence databases. Importantly, the interaction between AvrWTK4^{96224} and WTK4 or HMA+linker remains constant across different AlphaFold versions, and the interface does not change, although prediction confidence (ipTM) can vary slightly (see Supp. Note for more details).

It is increasingly recognised that AlphaFold predictions are often insensitive to interaction differences caused by single amino acid substitutions (Pak et al 2023, https://doi.org/10.1371/journal.pone.0282689; Wee et al. 2024, https://doi.org/10.1021/acs.jcim.4c00976). Of note, our experimental data show a reduction, but not a

complete loss of interaction when residues are mutated on the Avr surface (**Fig. 4D**). In this context, the observation that the AvrWTK4^{96224}-WTK4 interaction is consistently predicted with ipTM >0.6, whereas the avrWTK4$^{THUN\text{-}12}$-WTK4 prediction varies depending on the modelling setup, is broadly consistent with our experimental observations.

To ensure transparency and avoid overinterpretation, we have now included a detailed description of the modelling setups and results obtained with different AlphaFold versions in a new Supp. Note and propose to remove the original **Supp. Fig. S6**. We have accordingly revised the corresponding statements in the manuscript to avoid overinterpretation of the structural modelling results. In particular, we have toned down or removed statements suggesting that the modelling "confirms" the interaction, that the virulent Avr variant does not interact with WTK4, and also indicate that the modelling provides data consistent with the experimentally identified interface:

- Lines 339-345: "To test this, we used AlphaFold2 Multimer and AlphaFold3 at three different timepoints to model the complex (detailed description can be found in **Supp. Note**). The predicted ipTM value, indicative of model confidence, was on average 0.63 ~~0.73~~ for the AvrWTK4^{96224}-WTK4 (full length) in all settings and AlphaFold versions (lowest value: 0.51). ~~and 0.86 for~~ For the AvrWTK4^{96224}-HMA$_{WTK4}$ interaction, the predicted ipTM value was on average 0.84 (lowest value: 0.71), suggesting a stable association."
- Lines 351-358: "~~In fact, modelling the interaction between WTK4 and the virulent Avr variant, Bgt-55150$^{THUN\text{-}12}$ (hereafter, avrWTK4$^{THUN\text{-}12}$) yielded a much lower ipTM value of 0.17, indicating a loss of interaction (**Supp. Fig. S6**). This supports the hypothesis that only the avirulent AvrWTK4^{96224} variant stably interacts with HMA$_{WTK4}$, and that the mutations present in the virulent isolate likely disrupt this interaction.~~ The prediction of the interaction between full-length WTK4 and the virulent Bgt-55150$^{THUN\text{-}12}$ variant (hereafter, avrWTK4$^{THUN\text{-}12}$) showed variable results depending on the AlphaFold version and parameters used (**Supp. Note**). This may suggest a less stable interaction between the virulent avrWTK4 variant and WTK4."
- Lines 373-376: "These results were corroborated by yeast two-hybrid assays: strong interaction was observed between AvrWTK4^{96224} and HMA+linker, while the virulent avrWTK4$^{THUN\text{-}12}$ variant showed considerably weaker interaction (-WLH; **Fig. 4E**). This weak interaction is also consistent with the variable interaction results predicted by AlphaFold (**Supp. Note**)."
- Lines 421-423: Structural modelling and interaction assays ~~confirmed~~ showed that this mutation ~~strongly~~ reduces interaction with the HMA domain of WTK4 (**Fig. 4**; **Supp. Note** ~~**Supp. Fig. S6**~~).
- Lines 530-532: "~~Here, we predicted and then experimentally confirmed a specific interaction between HMA$_{WTK4}$ and the avirulent AvrWTK4 variant, but not with the virulent variant, illustrating the potential of AlphaFold for *in silico* analysis of recognition specificity (Supp. Fig. S6).~~"

7. Line 242-245 (Fig. 3A-B): "These data provide evidence that the WTK4-induced immune response results in both HR and papilla formation, very similar to other Pm resistance genes such as Pm4, encoding a kinase fusion protein (Sánchez-Martín et al. 2021)." I doubt that papilla formation is a defence response specifically to WTK4-containing lines (or other Pm gene-mediated resistant wheat lines), and the data provided do not permit this conclusion. The data shown in Fig 3A and 3B only show individual plant-pathogen interaction sites and do not allow quantitative conclusions to be drawn. It is well documented that the formation of papillae in response to powdery mildew inoculation is commonly observed in virulent powdery mildew-host interactions.

We rephrased the mentioned sentence to address the reviewer's comment. We also rephrased the figure legend to clarify that the images only represent examples of individual infection sites:

- Lines 253-257: "In both *Ae. tauschii* lines, at least ~~70~~ 25% of the spores attempting to penetrate were blocked ~~at a similar rate~~ by HR ~~and papilla formation~~ at all three timepoints (**Fig. 3A**~~**-B**~~; **Supp. Fig. S2**). Papilla formation was also observed (**Fig. 3B**; **Supp. Fig. S2**). These data provide evidence that the *WTK4*-induced immune response results in ~~both~~ HR ~~and papilla formation~~, very similar to other *Pm* resistance genes such as *Pm4*, encoding a kinase fusion protein (Sánchez-Martín et al. 2021)."
- Lines 296-300: "~~At the microscopic level, inoculation of a WTK4-containing Ae. tauschii line with CHE_96224 results in~~ **(A)** Example of a cell undergoing a hypersensitive response (brown colouring, visualised using DAB staining). (**B**) Example of a cell with papilla formation (round structure indicated by a black arrow). Both panels (A) and (B) represent microscopic evaluations of individual infection sites in a WTK4- containing line infected with CHE_96224 one day after inoculation."

Minor points:

1. Ln 375: please tone down the statement that the apparent reduction in binding is "strong" or provide quantitative interaction assays (e.g. ITC). Western blotting is sufficient to claim that the effector protein levels are comparable, but quantitative differences in protein levels may contribute to the reduction in cell death activity.

We agree that the sentence is imprecise. We reformulated it, toning it down as suggested.

- Lines 421-422: "Structural modelling and interaction assays ~~confirmed~~ showed that this mutation ~~strongly~~ reduces interaction with the HMA domain of WTK4"

2. Line 194: "encoding an ankyrin-repeat-containing protein". This needs to be corrected to "encoding a deduced ankyrin-containing protein" throughout the manuscript.

We changed "ankyrin-repeat-containing protein" into "deduced ankyrin-repeat-containing protein" throughout the manuscript (lines 118-119, 206-207, 430-431).

3. It is a common misconception that the split-luciferase assay detects protein-protein interactions in vivo, as the analysis involves tissue destruction and it remains unclear whether the protein interactions occur only after cell homogenization. This must be changed in line 315 and throughout the manuscript.

Thank you for pointing this out. We changed the text so that it does not refer anymore to the split luciferase experiment results as "*in vivo*" but rather "*in planta*", following your minor comment nr. 5 (line 359).

4. The reference list is in parts outdated and lacks a number of recent publications describing high resolution structures of RNase-like powdery mildew avirulence effectors and the structure of a heterocomplex of such an avirulence effector with its matching sensor NLR. These are relevant for a

more general interpretation and discussion of the diversity of RNase-like effector interactions with host components.

We agree that the reference list was partially outdated and lacked several recent relevant publications. We have now included recent studies describing high-resolution structures of RNase-like powdery mildew avirulence effectors, as well as the structure of an effector-NLR heterocomplex. In addition, we incorporated newly available preprints on HMA-like domains, which are highly relevant to this study. We also updated references that were previously cited as bioRxiv preprints and have since been published. Finally, we corrected our original statement that all Avr proteins identified in *Bgt* exhibit an RNase-like fold, in light of recent work by Kunz et al. (2026), which identified an Avr with a different structural fold.

After inclusion of the new references, the following parts of the manuscript have been modified:

- Lines 85-87: "Although effectors from *Bgt* and *Bh* display high sequence diversification, the RNase-like structure is highly conserved in these species (Cao et al. 2023)."
- Line 87-88: "So far, the only NLR-recognised Avr effector in *Bgt* that does not have an RNase-like structure is the recently described AvrPm3e_1 (Kunz et al. 2025)."
- Line 96-97: "In *Bh*, Avra effectors directly interact with their corresponding Mla receptors (Saur, Bauer, Kracher, et al. 2019; Lawson et al. 2025)."
- Line 315: "AvrWTK4^{96224} is predicted to exhibit the typical RNase-like fold of most ~~all~~ *Bgt Avrs* identified to date".
- Lines 331-333: "We found that RPG1, the first TKP identified in barley (Brueggeman et al. 2002), also has an HMA-like ID at its N-terminus (52.9% sequence identity with HMA_{WTK4} at the protein level, and 93% at the structural level; **Supp. Fig. S4, Table 1**), as recently described by Reveguk et al. (2025). A recent study described the cloning of *Rmo2* and *Rwt7*, resistance genes against *M. oryzae*, encoding TKPs which also possess HMA-like integrated domains (Asuke et al. 2025)."
- Lines 492-494: "Moreover, the presence of IDs in at least ~~four~~ six characterised resistance proteins with a TKP structure (WTK4, RPG1, Lr9, and RWT4, Rmo2, Rwt7; this study; Sung et al., 2025; Wang et al., 2023~~)~~(, Asuke et al. 2025), suggests a common, functional role in pathogen recognition."
- Lines 527-529: "A recent study presented the targeted engineering of the HMA-like domain of the tandem kinase protein Rmo2 to recognise the wheat blast effector PWT7, in addition to the naturally recognised PBY2, highlighting the potential of this approach in TKPs (Yu et al. 2025)."

We added/modified the following references (they are underlined in the manuscript and in the bibliography):

- Bernasconi, Z., Herger, A. G., Caro, M. D. P., Kunz, L., Müller, M. C., Stirnemann, U., … Keller, B. (2026). Virulence on Pm4 kinase-based resistance is determined by two divergent wheat powdery mildew effectors. *Nature Plants*, *12*(1), 164–178. https://doi.org/10.1038/s41477-025-02180-w (cited multiple times throughout the manuscript; this entry substitutes the preprint version)
- Cao, Y., Kümmel, F., Logemann, E., Gebauer, J. M., Lawson, A. W., Yu, D., … Schulze-Lefert, P. (2023). Structural polymorphisms within a common powdery mildew effector scaffold as a driver of coevolution with cereal immune receptors. *Proceedings of the National Academy of Sciences*, *120*(32), 1–11. https://doi.org/10.1073/pnas.2307604120
- Kunz, L., Bernasconi, Z., Heuberger, M., Isaksson, J., Sotiropoulos, A. G., Stirnemann, U., … Keller, B. (2025). Dual recognition of structurally unrelated mildew effectors underlies the broad-spectrum resistance of Pm3e in wheat. *BioRxiv*. https://doi.org/10.1101/2025.10.26.683672
- Lawson, A. W., Flores-Ibarra, A., Cao, Y., An, C., Neumann, U., Gunkel, M., … Schulze-Lefert, P. (2025). The barley MLA13-AVRA13 heterodimer reveals principles for immunoreceptor recognition

of RNase-like powdery mildew effectors. *The EMBO Journal*, *44*(11), 3210–3230. https://doi.org/10.1038/s44318-025-00373-9

- Asuke, Soichiro, Analiza G. Tagle, Gang-Su Hyon, Sayako Koizumi, Tsubasa Murakami, Akiko Horie, Daisuke Niwamoto, et al. (2025). Evolution of HMA-Integrated Tandem Kinases Accompanied by Expansion of Target Pathogens. *BioRxiv*. https://doi.org/10.64898/2025.12.15.692859
- Yue, N., Liu, D., & Liu, Y. (2025). Tandem kinase–NLR pairs: Novel paradigms in cereal disease resistance. *Plant Communications*, *6*(8), 101435. https://doi.org/10.1016/j.xplc.2025.101435
- Yu, Daniel S, Rafał Zdrzałek, Emi Katayama, Hitomi Akiyama, Lucy Daykin, Nathan J Williams, Iwan Goodridge, Soichiro Asuke, and Mark J Banfield. (2025). Engineering Plant Tandem Kinase Immune Receptors Expands Effector Recognition Profiles. *BioRxiv*. https://doi.org/10.64898/2025.12.15.694194

5. It is a widespread misconception that the described luciferase assay detects in vivo protein-protein interactions because the analysis involves tissue disruption and it remains unclear whether the protein interactions occur after cellular disintegration. This needs to be changed in l. 315 and throughout the manuscript. It's an in planta assay, but not an in vivo assay.

As stated above (minor comment nr. 3), we changed the text so that it does not refer anymore to the split luciferase experiment results as "in vivo" (only occurrence in line 359).

Revisions to manuscript NCOMMS-25-71472B:

An HMA-like integrated domain in the wheat tandem kinase WTK4 recognises an RNase-like pathogen effector.

Dear Editors and Reviewers,

Thank you very much for your evaluation of our response to your comments and suggestions. We are pleased with the feedback on our revised manuscript.

We have addressed the remaining comments from Reviewer #2, as detailed in the point-by-point response below. The corresponding changes have been incorporated into the revised manuscript. All modifications made during the first round of revisions (previously highlighted using track changes) have been accepted, and only the new changes made in response to Reviewer #2's latest comments are now visible in track change mode. The revised Word document has been uploaded to the submission platform.

Additionally, we have updated the reference formatting style to align with the style required by *Nature Communications*.

Thank you for considering this revised version for publication in *Nature Communications*.

Best regards,

The authors

Reviewer #1 (Remarks to the Author):

The revised manuscript made significant revision based on the comments of the reviewers. The authors performed additional analysis and experiments, and provided strong new information to support their findings. I am satisfied with the scientific meaning and content of the revision. I have no further comments for the revised manuscript.

We thank the reviewer for the positive feedback and constructive comments.

Reviewer #2 (Remarks to the Author):

I am largely satisfied with the author's revision. They have conducted additional experiments that strengthen their claims (e.g. testing co-expression of WTK4 and AvrWTK496224 in one additional Ae. tauschii line; testing virulent Avr variant Bgt-55150AvrWTK4_3 and the non-recognized effector AvrPm3b2/c2). Additionally, they eliminated statements that AF 'confirmed' some of the modelled interaction interfaces. The new Supplementary Table SN1 provides more detail on the modelling and acknowledges the variability. It is now clearer that AF cannot distinguish between WTK4 interactions with virulent and avirulent variants of the corresponding effectors.

We thank the reviewer for the positive feedback and constructive comments.

Two points remain to be fixed:

1. Line 419ff:"Strikingly, two independent AvrWTK4 mutants have the exact same mutation in Bgt-55150, resulting in the T30N substitution at the protein level, a mutation that is also present in the naturally virulent isolate THUN-12. Structural modelling and interaction assays showed that this mutation strongly reduces interaction with the HMA domain of WTK4 (Fig. 4; Supp. Note).“

Neither the structural modelling, nor the interaction assays were performed with a AvrWTK4 variant carrying only a T30N substitution (e.g. AvrWTK4-3), but with AvrWTK4THUN12 which contains two additional aa polymorphisms. Thus, there is no data to support that this mutation specifically affects the interaction with the HMA domain of WTK4. Furthermore, as the authors state, AF modelling cannot yet distinguish between potential differences in protein interactions caused by single amino acid substitutions. Therefore, structural modelling is an inappropriate method with which to assess the impact of T30N on interaction.

We agree that this statement in our discussion section is not supported sufficiently by the presented data, and we apologise for the incorrect sentence. We modified it as follows:

New lines 393-399: "Strikingly, two independent *AvrWTK4* mutants have the exact same mutation in *Bgt-55150*, resulting in the T30N substitution at the protein level, a mutation that is also present in the naturally virulent isolate THUN-12, suggesting its importance in evasion from recognition ~~Structural modelling and interaction assays showed that this mutation reduces interaction with the HMA domain of WTK4~~ (**Fig. 4**; **Supp. Note**). Other studies, mostly done with NLR immune receptors, have shown that single point mutations in pathogen effectors allow escape from recognition. For instance, the globally widespread AvrPm8_F43Y variant effectively evades recognition by Pm8 [24]."

2. In Supp Table SN1, the forward slash „/“ is missing between 0.482 0.292.

Thank you for noticing this mistake. We found the same typo in Table SN2 (between 0.71 and 0.69) and corrected both. Additionally, we corrected two other typos found in the Supplementary Note (a repetition of “score” in line 4, and “PyMOL” instead of “Pymol” in line 44).
