# Supplementary material for: An HMA-like integrated domain in the wheat tandem kinase WTK4 recognises an RNase-like pathogen effector
Source: Nat Commun. 2026 Jul 6;17:8458. doi: 10.1038/s41467-026-74940-x (PMC13478195; doi:10.1038/s41467-026-74940-x)
Supplement: Supplementary file 3 [file 41467_2026_74940_MOESM1_ESM.pdf]

Supplementary Figures

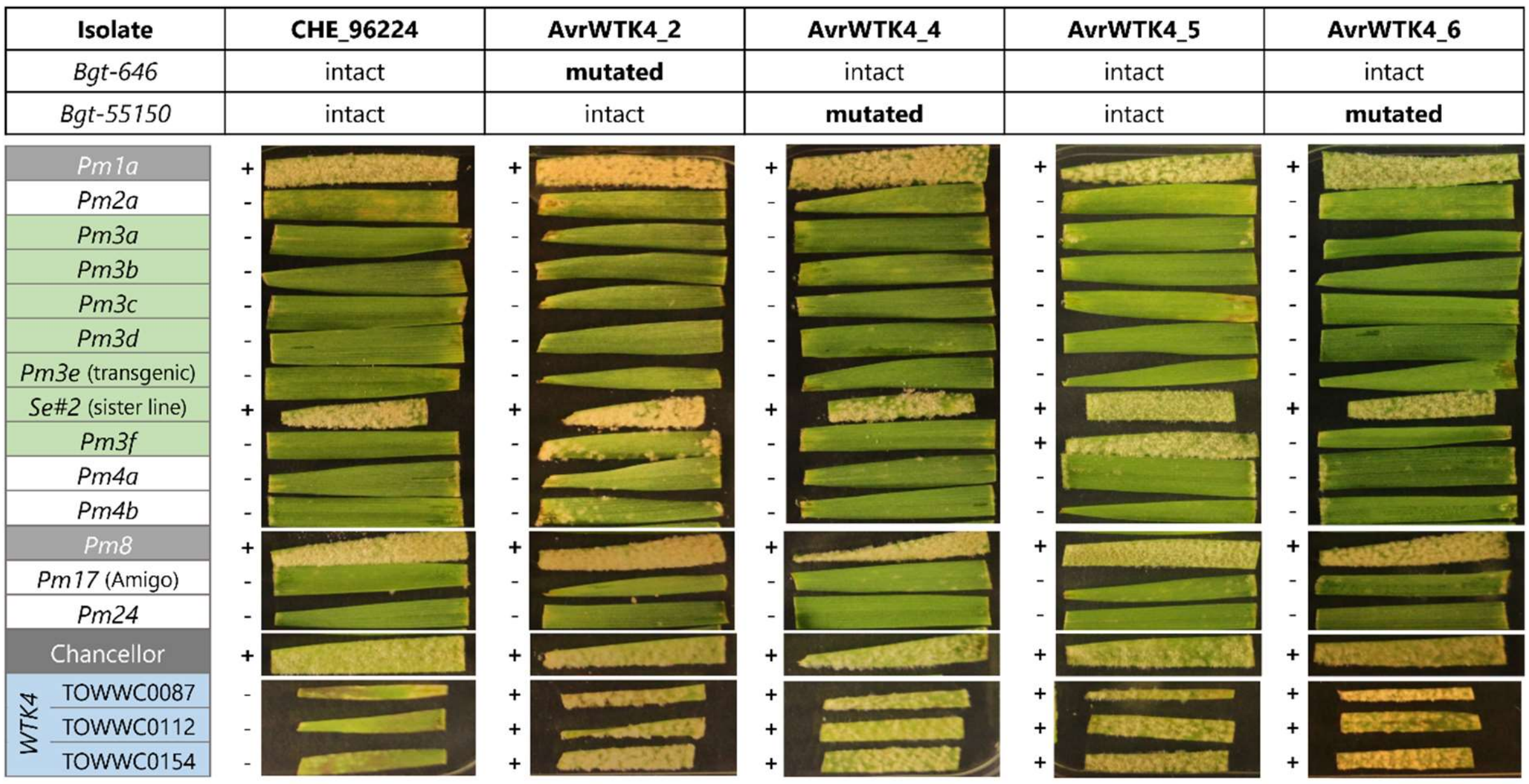

Supplementary Fig. 1. ***AvrWTK4* mutants are virulent on *WTK4*-containing lines**. The *AvrWTK4* mutants exhibit specific gain of virulence on three different *Ae. tauschii* lines (TOWWC0087, TOWWC0112, and TOWWC0154), but no virulence on near-isogenic lines (NILs) carrying various *R* genes for which their parental isolate CHE_96224 is avirulent. The exception is mutant AvrWTK4_5, which exhibits additional gain of virulence on *Pm3f*, caused by an unknown mutation. Cultivars susceptible to CHE_96224 (the *Pm1a* NIL, the sister line of the transgenic *Pm3e* line (here called *Se#2*), the *Pm8* NIL and Chancellor) are highlighted in grey. NILs containing *Pm3* alleles are depicted in green, and the three *Ae. tauschii* lines containing *WTK4* are in blue. The remaining *Pm* lines are shown in white. The symbol on the left side of every column indicates a virulent (+) or an avirulent (-) phenotype.

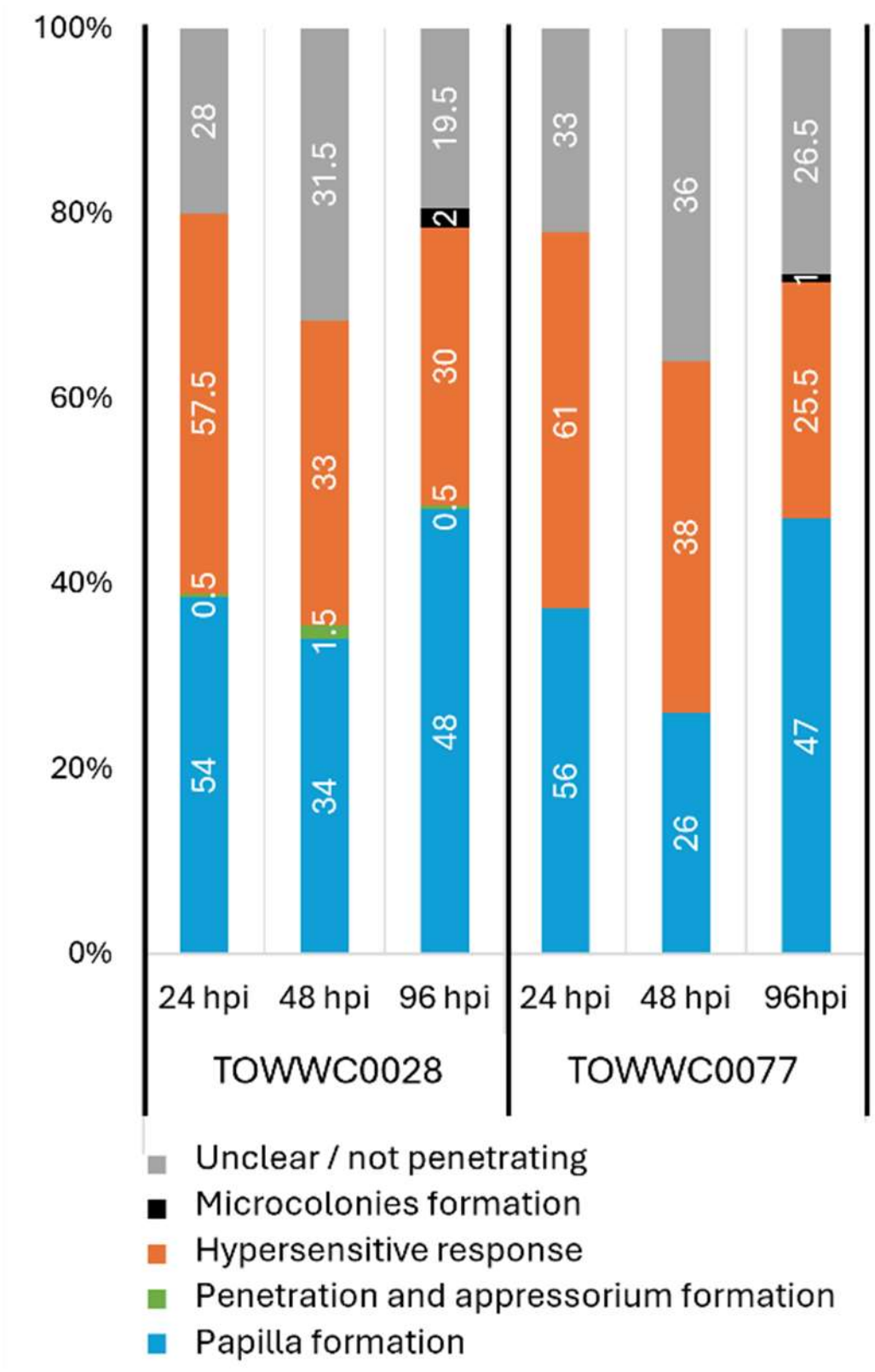

Supplementary Fig. 2. Microscopic evaluation of the immune response induced by the avirulent *Bgt* isolate CHE_96224 on two *WTK4*-containing *Ae. tauschii* lines (TOWWC0028 and TOWWC0077). At least 100 spores per leaf, time point, and line were visually inspected. Coomassie staining was used to visualise fungal structures (blue/purple), and DAB staining was used to identify cells undergoing hypersensitive response (HR) (dark brown). The number of *Bgt* spores causing HR (orange in the graph), blocked by papilla formation (blue), penetrating and creating an appressorium (green) or forming a microcolony (black) were counted. If the reaction was unclear, spores were classified as “Unclear / not penetrating”. Examples of a *Bgt* spore attempting to penetrate a plant cell undergoing HR and of a *Bgt* spore attempting to penetrate a plant cell blocked by papilla formation are shown in **Fig. 3A-B**, respectively.

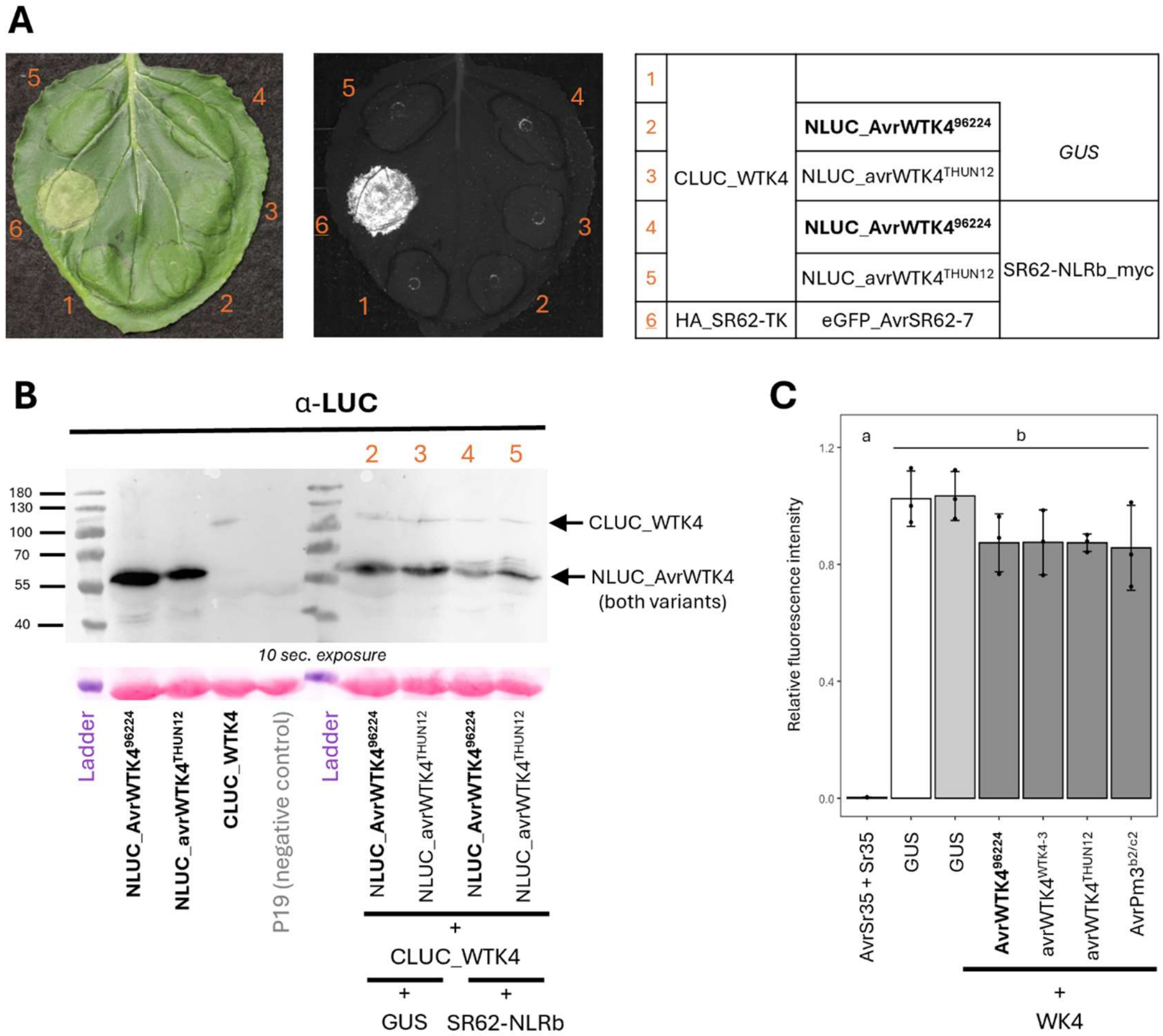

Supplementary Fig. 3. Co-expression of *AvrWTK4*^{96224} and *WTK4* does not lead to cell death in *N. benthamiana* or wheat protoplasts (wheat cv. Fielder). (A) CLUC_WTK4 was co-infiltrated in *N. benthamiana* either alone, with NLUC_AvrWTK4^{96224} or NLUC_avrWTK4$^{THUN-12}$ (infiltrations 1-3). In infiltrations 4 and 5, SR62^{NLR} was added to CLUC_WTK4 and either NLUC_AvrWTK4^{96224} or NLUC_avrWTK4$^{THUN-12}$. As positive control, SR62^{NLR} was co-infiltrated with SR62^{TK} and AvrSR62-7 (infiltration 6). No cell death was observed for any infiltration except for the positive control. The cell death signal was measured 5 days post-infiltration. **(B)** Immunoblot analysis of WTK4 and AvrWTK4 constructs used in the cell death assay shown in panel A. Protein samples were prepared as described in the Methods section and separated on a 10% SDS-polyacrylamide gel. On the left side of the membrane, proteins from single infiltrations (2:1 construct:P19 ratio) are shown. On the right side, proteins from co-infiltrations (1:1:1 AVR:R:P19 ratio) are shown. The membrane was incubated with an anti-LUC antibody and imaged for 10 seconds **(C)** The YFP fluorescence in wheat protoplasts was measured 18 hours post-transfection and normalised to the fluorescence value of protoplast transfected with YFP with GUS. As a positive control, *Sr35* co-transfected with its corresponding *Avr* effector *AvrSr35* was used. Two technical and three biological replicates were measured per transfection. Significance and p values (significance threshold of $p < 0.05$) were calculated using a two-way analysis of variance (ANOVA) followed by a Tukey's HSD test (**Supplementary Data 7**). Bars with the same letter are not significantly different.

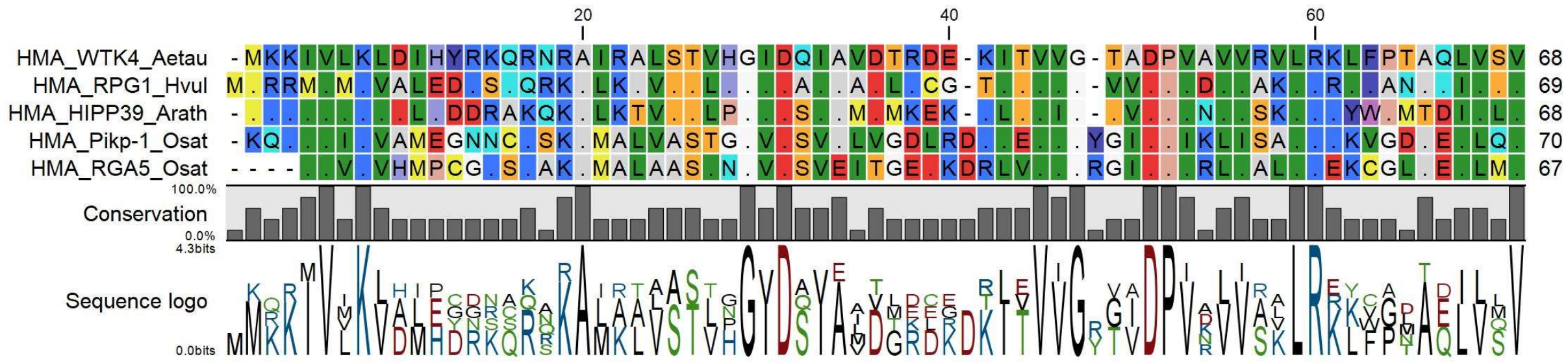

Supplementary Fig. 4. **Protein alignment of the HMA domains of WTK4 (from *Ae. tauschii*), RPG1 (from barley), Pikp-1, RGA5 (both from rice), and HIPP39 (the closest HMA_{WTK4} domain homologue from *Arabidopsis thaliana*)**. The amino acid colouring reflects traditional biochemical properties of the amino acids (e.g. polarity) and shows a high degree of conservation between the different HMA domains. The HMA domain sequences were extracted from the UniProt database or by blasting the HMA domain of WTK4 in the NCBI database and aligned using Clustal Omega.

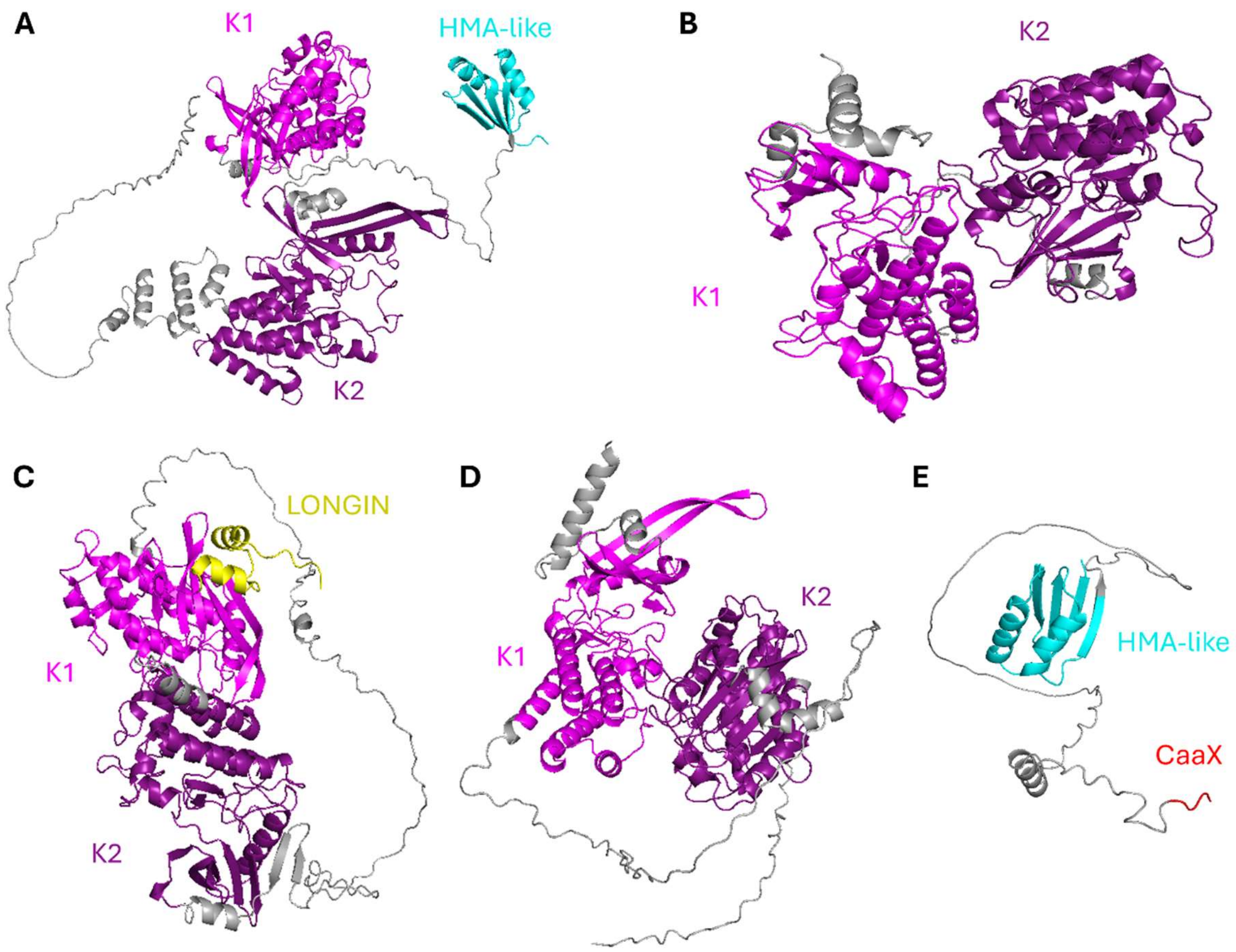

Supplementary Fig. 5. **Protein structure prediction of TKPs and the HMA-domain containing protein HIPP39.** (**A**) RPG1, (**B**) Yr15 (WTK1), (**C**) Sr60 (WTK2), (**D**) Sr62^{TK} (WTK5) and (**E**) HIPP39. The 3D structure prediction for other tandem kinase proteins, i.e. WTK6-vWA, WTK7-TM and RWT4 (allelic to Pm24) can be found in the studies of Wang et al. (2023), Li et al. (2024) and Sung et al. (2025), respectively. The HMA-like domains are shown in cyan and consist of the typical βαββαβ 3D structure. The two kinase domains of the tandem-kinase proteins (TKPs) are indicated as K1 (light magenta) and K2 (dark magenta) based on their order (from the N-terminus) in the protein. K1 is a pseudokinase in WTK4 and RPG1, whereas it is an active kinase in all other TKPs. K2 is an active kinase for WTK4, RPG1 and Sr60 (which has two active kinases), and a pseudokinase for the other WTKs. In addition, Sr60 has a predicted N-terminal LONGIN domain, possibly involved in association with vesicular membrane proteins (in yellow, predicted with low confidence by ScanProsite). HIPP39 has a C-terminal isoprenylation motif (CaaX, in red; A being an aliphatic amino acid, and X any amino acid). The linker sequences (without any domain prediction) between the different domains are shown in grey. All structures have been predicted with AlphaFold2 and visualised with PyMOL.

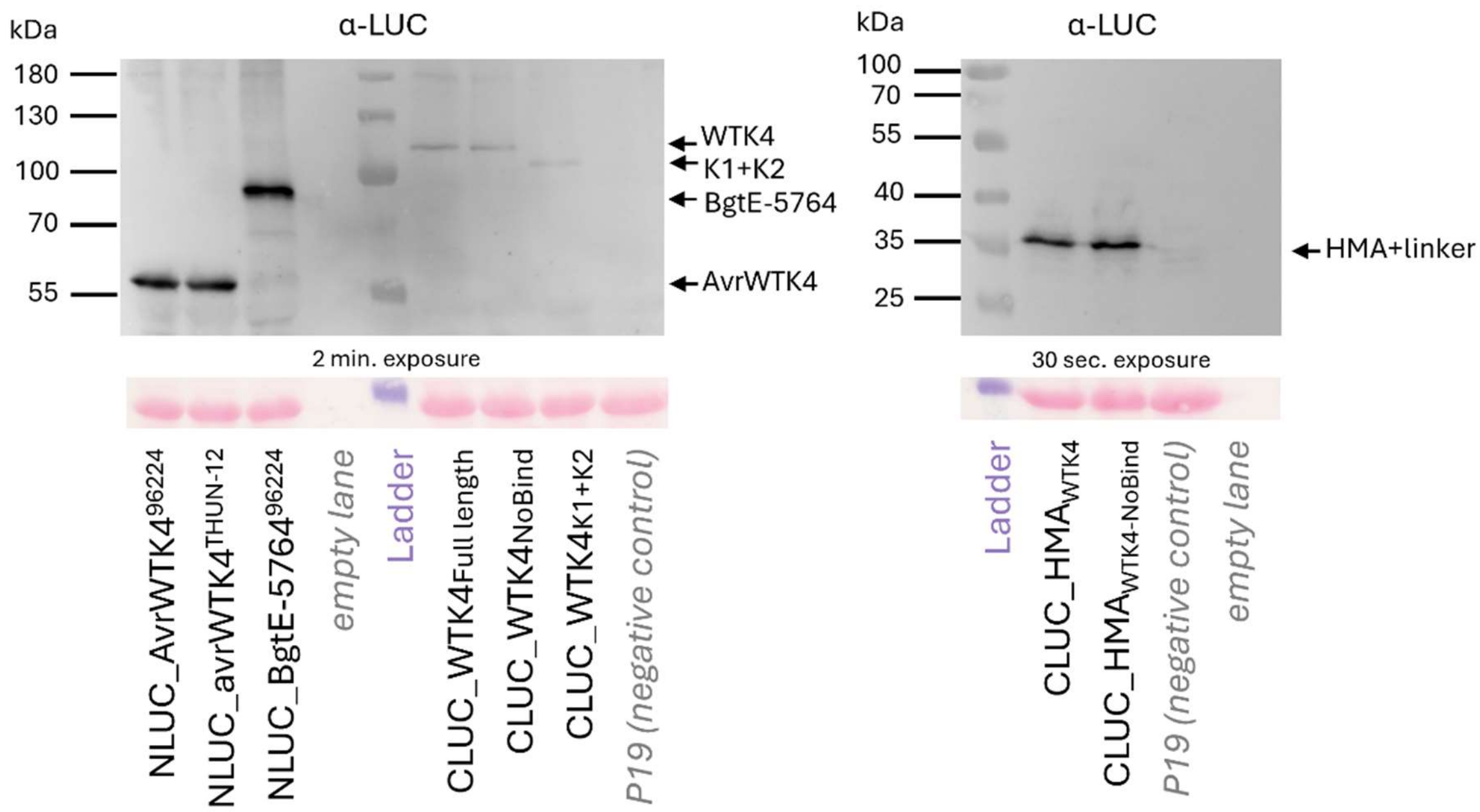

Supplementary Fig. 6. Protein detection of the proteins used in the split-luciferase experiment. LUC-tagged constructs used in the split-luciferase assay were infiltrated into *Nicotiana benthamiana* leaves along with P19, using a construct-to-P19 ratio of 2:1. Three days after infiltration, six leaf discs were collected from three different leaves, and total protein was extracted as outlined in the Methods section. The Avr, WTK4 full-length and K1+K2 constructs were loaded on a 7.5% SDS-polyacrylamide gel, while the HMA+linker constructs on a 12% SDS-polyacrylamide gel. The membrane containing the Avrs, WTK4 full-length and K1+K2 constructs was exposed for 2 minutes, while the membrane containing the HMA+linker constructs for 30 seconds. Protein levels were comparable between WTK4 and $WTK4_{NoBind}$, HMA_{WTK4} and $HMA_{WTK4\text{-}NoBind}$, and $AvrWTK4^{96224}$ and $avrWTK4^{THUN\text{-}12}$. Below, the membranes were stained with a Ponceau solution as a loading control.

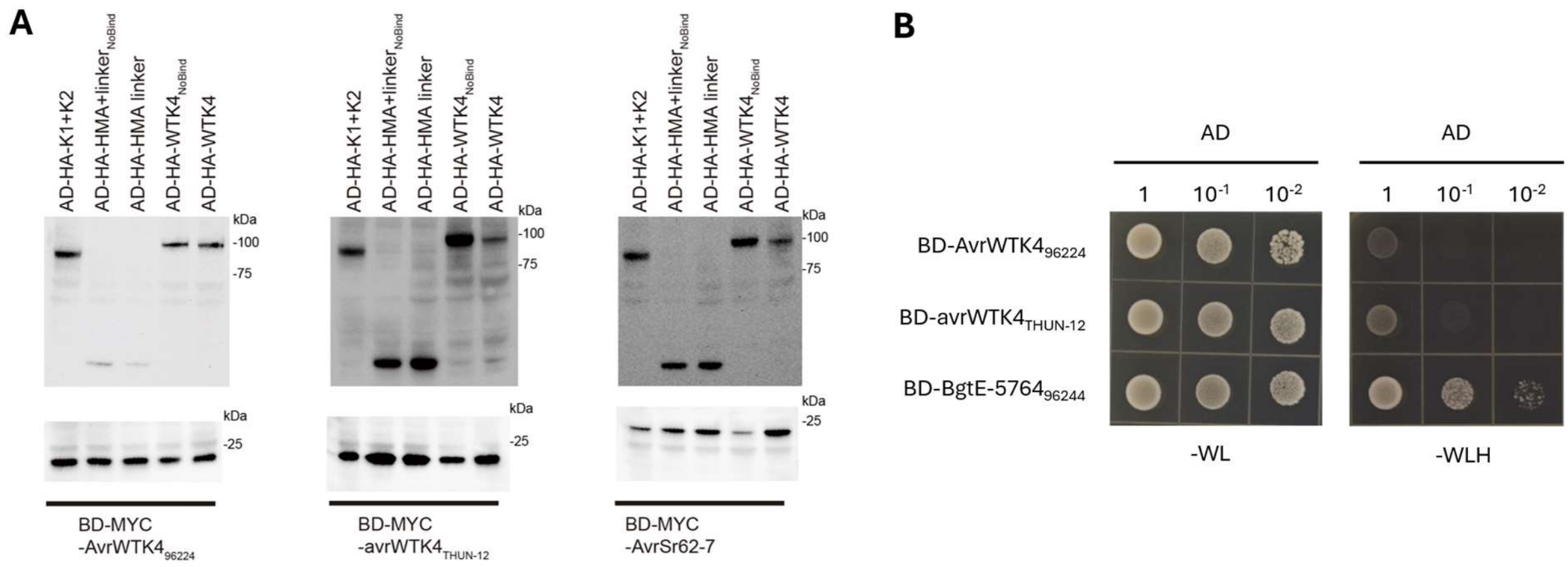

Supplementary Fig. 7. **Protein detection of yeast two-hybrid constructs and autoactivity of BgtE-5764.** (**A**) Immunoblot detection of protein expressed in yeast in **Fig. 4E**, detected using anti-HA or anti-Myc antibodies. The coding sequences of the HA and Myc tags are integrated into the AD and BD vectors, respectively, and are in the same reading frame as their corresponding fusion proteins. (**B**) The BD-BgtE-5764^{96224} fusion protein exhibited autoactivation activity in yeast. BD-BgtE-5764^{96224} was co-transformed with the empty vector pGADT7 into the yeast strain AH109, it grew normally on the selective medium SD-3, indicating that the BgtE-5764^{96224} fusion protein is unsuitable for use as a negative control in yeast two-hybrid experiments.

Supplementary Note: AlphaFold predictions of interactions

For the AlphaFold-based predictions of the interaction between AvrWTK4 variants and WTK4, different software versions were tested. **Supp. Tables SN1-SN2** show the predicted pTM (predicted Template Modeling) and ipTM (interface predicted Template Modeling) scores for AvrWTK4^{96224} and avrWTK4$^{THUN\text{-}12}$, either alone or in combination with WTK4 (full-length) or the HMA+linker domain of WTK4 (amino acids 1-104). pTM indicates the confidence in the overall predicted protein structure, whereas ipTM estimates the confidence of the predicted interaction interface between proteins. Both Avr variants were predicted as full-length proteins including the signal peptide (SP) (labelled "full-SP"), as proteins with a truncated SP containing only 10 amino acids ("SP-10"), and as proteins without the SP ("no-SP"). AlphaFold2 (**Supp. Table SN1**) and AlphaFold3 (**Supp. Table SN2**) were used to compare prediction performance, with AlphaFold3 being tested at three different time points. AlphaFold2 predictions were performed using the ColabFold implementation with default parameters (v1.5.5) and AlphaFold3 predictions were performed via the AlphaFold3 server (https://alphafoldserver.com/). In July 2025, only a subset of the interactions was tested (**Supp. Table SN2i**). Some differences were observed in AlphaFold3 predictions across different time points, likely reflecting updates to the AlphaFold3 model and underlying sequence databases (**Supp. Table SN2**).

Predictions of the effectors without the SP, either alone or in combination with WTK4 or HMA+linker, were consistently poorer and led to overall lower pTM and ipTM values than predictions including the full SP (**Supp. Tables SN1-SN2**). Predictions of AvrWTK4 variants with a truncated SP (SP-10) gave variable results (**Supp. Tables SN1-SN2**). Since the SP was never involved in the predicted interaction interface, and its presence improved prediction quality, SP-containing sequences were used for all subsequent analyses. The SP was then manually removed in PyMOL.

A large difference in ipTM score between the AvrWTK4^{96224}-WTK4 and avrWTK4$^{THUN\text{-}12}$-WTK4 interactions was observed in predictions by AlphaFold2 and AlphaFold3 in July 2025 (highlighted in bold in **Supp. Tables SN1** and **SN2i**). However, this difference was much smaller or absent in AlphaFold3 predictions generated in October 2025 and January 2026 **(Supp. Table SN2ii-iii**). In contrast, the AvrWTK4^{96224}-HMA+linker and avrWTK4$^{THUN\text{-}12}$-HMA+linker interactions were consistently predicted with high ipTM scores by both software versions at all time points.

Importantly, for both AlphaFold2 and AlphaFold3 (at all tested time points), the predicted interaction surface between AvrWTK4^{96224} and WTK4 was identical, involving the same residues of the HMA+linker domain and the Avr protein (indicated by the arrow and asterisk in **Supp. Fig. SN1**). The residues involved in the predicted interaction (amino acids 29 to 34, for both AvrWTK4 and WTK4) had consistently high pLDDT values (>68 for AvrWTK4 and >79 for WTK4), indicative of high structural confidence (**Supp. Table SN3**).

Supp. Table SN1. Predictions by AlphaFold2 (ColabFold v1.5.5)

Date: 14.01.2026	Avr pTM	Avr - WTK4 pTM / iPTM	Avr - HMAlinker pTM / iPTM
AvrWTK4^{96224}_no-SP	0.509	0.459 / 0.238	0.477 / 0.375
AvrWTK4^{96224}_SP-10	0.743	0.513 / 0.787	0.85 / 0.891
AvrWTK4^{96224}_full-SP	0.71	0.466 / **0.73**	0.803 / 0.901
avrWTK4$^{THUN-12}$_no-SP	0.545	0.459 / 0.235	0.447 / 0.304
avrWTK4$^{THUN-12}$_SP-10	0.634	0.486 / 0.245	0.618 / 0.649
avrWTK4$^{THUN-12}$_full-SP	0.704	0.482 / **0.292**	0.804 / 0.901

Supp. Table SN2. Predictions by AlphaFold3 (Alphafold server)

i. Date: 22. / 29.07.2025	Avr pTM	Avr - WTK4 pTM / iPTM	Avr - HMAlinker pTM / iPTM
AvrWTK4^{96224}_no-SP	n.a.	n.a.	n.a.
AvrWTK4^{96224}_SP-10	n.a.	n.a.	n.a.
AvrWTK4^{96224}_full-SP	n.a.	0.55 / **0.62**	0.72 / 0.71
avrWTK4$^{THUN-12}$_no-SP	n.a.	n.a.	n.a.
avrWTK4$^{THUN-12}$_SP-10	n.a.	n.a.	n.a.
avrWTK4$^{THUN-12}$_full-SP	n.a.	0.53 / **0.17**	0.71 / 0.69

ii. Date: 13.10.2025	Avr pTM	Avr - WTK4 pTM / iPTM	Avr - HMAlinker pTM / iPTM
AvrWTK4^{96224}_no-SP	0.79	0.44 / 0.28	0.86 / 0.87
AvrWTK4^{96224}_SP-10	0.53	0.54 / 0.48	0.86 / 0.88
AvrWTK4^{96224}_full-SP	0.72	0.45 / **0.66**	0.8 / 0.88
avrWTK4$^{THUN-12}$_no-SP	0.79	0.43 / 0.22	0.87 / 0.89
avrWTK4$^{THUN-12}$_SP-10	0.75	0.45 / 0.68	0.84 / 0.88
avrWTK4$^{THUN-12}$_full-SP	0.72	0.44 / **0.62**	0.8 / 0.89

iii. Date: 14.01.2026	Avr pTM	Avr - WTK4 pTM / iPTM	Avr - HMAlinker pTM / iPTM
AvrWTK4^{96224}_no-SP	0.8	0.45 / 0.15	0.85 / 0.86
AvrWTK4^{96224}_SP-10	0.52	0.48 / 0.62	0.78 / 0.79
AvrWTK4^{96224}_full-SP	0.73	0.44 / **0.51**	0.79 / 0.86
avrWTK4$^{THUN-12}$_no-SP	0.79	0.49 / 0.14	0.87 / 0.89
avrWTK4$^{THUN-12}$_SP-10	0.45	0.44 / 0.15	0.84 / 0.89
avrWTK4$^{THUN-12}$_full-SP	0.72	0.43 / **0.65**	0.8 / 0.89

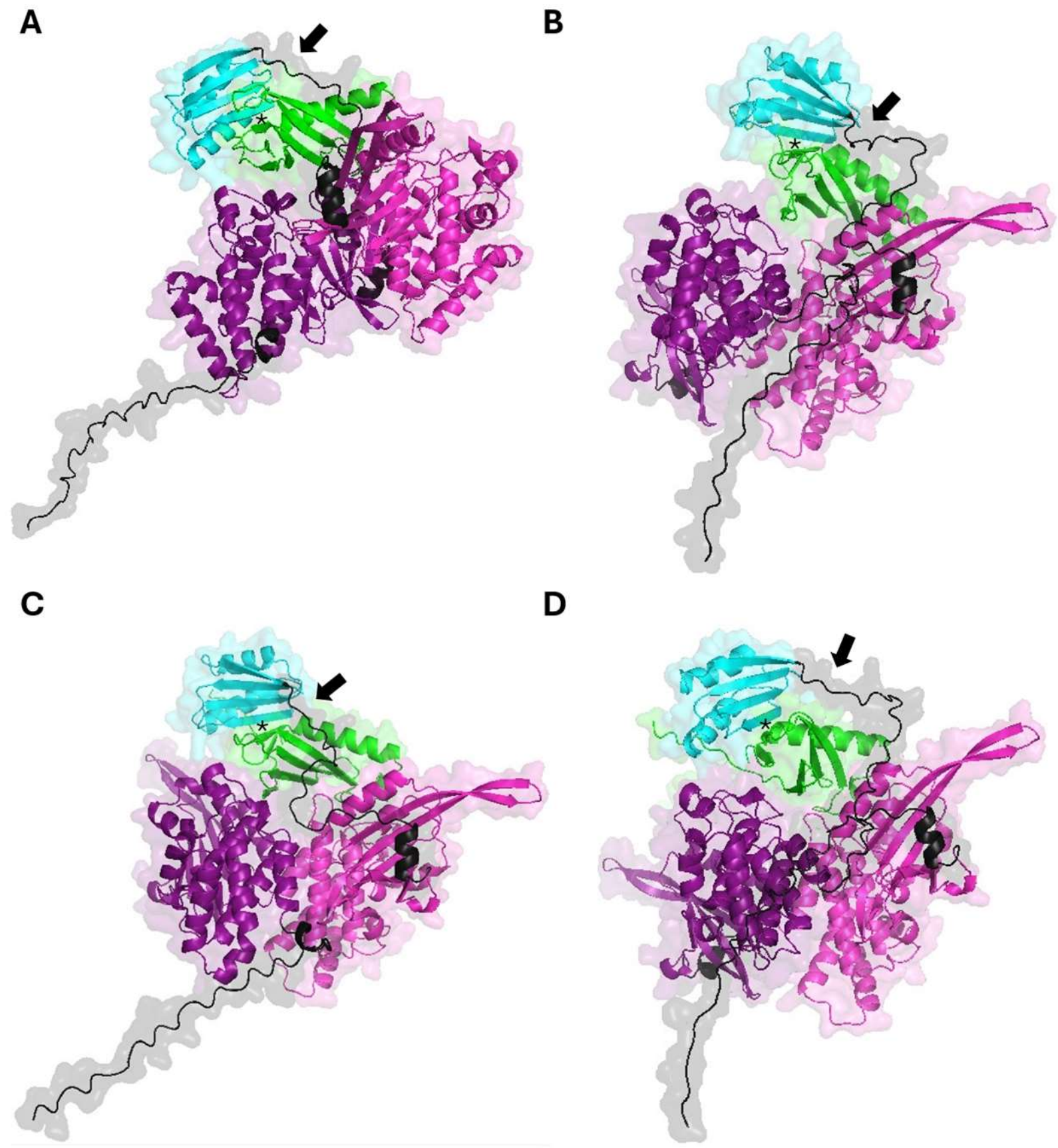

Supp. Fig. SN1. Prediction of the WTK4-AvrWTK4^{96224} interaction using (A) AlphaFold2, (B) AlphaFold3_July2025, (C) AlphaFold3_October2025 and (D) AlphaFold3_January2026. The interaction surface is the same for all predictions. The structures were visualised in PyMOL.

Supp. Table SN3. pLDDT per residue of the predicted AvrWTK4^{96224} and WTK4 main interaction surface.

AvrWTK4 residue		AlphaFold2 January 26	AlphaFold3 July 25	AlphaFold3 October 25	AlphaFold3 January 26
29	E	68.12	75.64	77.14	73.44
30	T	73.12	81.2	81.02	76.1
31	Y	78.44	83.02	83.8	76.12
32	I	79.5	84.92	85.91	79.42
33	T	76	84.99	85.09	78.67
34	V	72.88	85.69	85.99	81.45
**WTK4 residue**		**AlphaFold2 January 26**	**AlphaFold3 July 25**	**AlphaFold3 October 25**	**AlphaFold3 January 26**
29	I	87.62	87.34	88.5	84.14
30	D	86.75	86.46	87.66	82.38
31	Q	87.05	88.33	88.94	84.43
32	I	86.25	87.48	88.38	84.06
33	A	83.19	86.1	86.83	84.7
34	V	79.56	82.57	84.47	81.66
