# Supplementary material for: An HMA-like integrated domain in the wheat tandem kinase WTK4 recognises an RNase-like pathogen effector
Source: Nat Commun. 2026 Jul 6;17:8458. doi: 10.1038/s41467-026-74940-x (PMC13478195; doi:10.1038/s41467-026-74940-x)
Supplement: Supplementary file 4 [file 41467_2026_74940_MOESM2_ESM.pdf]

## Descriptions of Additional Supplementary Files

**Supplementary Data1.** Detection of WTK4 in Ae. tauschii lines and their corresponding phenotypes

**Supplementary Data 2.** Phenotyping of selected F1 progeny of the CHE_96224 x THUN-12 cross on three WTK4 Ae. tauschii lines

**Supplementary Data 3.** Description of the genes contained in the AvrWTK4 locus

**Supplementary Data 4.** Mutations present in the AvrWTK4 mutants across the genome

**Supplementary Data 5.** Description of gene-proximal variants (GPV) and the corresponding affected genes

**Supplementary Data 6.** Primers used in this study

**Supplementary Data 7.** Statistical analyses of RTqPCR, split-luciferase, and protoplast experiments

**Supplementary Data 8.** Constructs used for cell death and protein-protein interaction assays
